# PAGA: graph abstraction reconciles clustering with trajectory inference through a topology preserving map of single cells

**DOI:** 10.1186/s13059-019-1663-x

**Published:** 2019-03-19

**Authors:** F. Alexander Wolf, Fiona K. Hamey, Mireya Plass, Jordi Solana, Joakim S. Dahlin, Berthold Göttgens, Nikolaus Rajewsky, Lukas Simon, Fabian J. Theis

**Affiliations:** 1 Helmholtz Center Munich – German Research Center for Environmental Health, Institute of Computational Biology, Neuherberg, Munich, Germany; 2 Department of Haematology and Wellcome and Medical Research Council Cambridge Stem Cell Institute, University of Cambridge, Cambridge, UK; 3 Berlin Institute for Medical Systems Biology, Max-Delbrück Center for Molecular Medicine, Berlin, Germany; 4 Department of Medicine, Karolinska Institutet and Karolinska University Hospital, Stockholm, Sweden; 5 Department of Mathematics, Technische Universität München, Munich, Germany

## Abstract

Single-cell RNA-seq quantifies biological heterogeneity across both discrete cell types and continuous cell transitions. Partition-based graph abstraction (PAGA) provides an interpretable graph-like map of the arising data manifold, based on estimating connectivity of manifold partitions (https://github.com/theislab/paga). PAGA maps preserve the global topology of data, allow analyzing data at different resolutions, and result in much higher computational efficiency of the typical exploratory data analysis workflow. We demonstrate the method by inferring structure-rich cell maps with consistent topology across four hematopoietic datasets, adult planaria and the zebrafish embryo and benchmark computational performance on one million neurons.

**Electronic supplementary material:**

The online version of this article (10.1186/s13059-019-1663-x) contains supplementary material, which is available to authorized users.

## Background

Single-cell RNA-seq offers unparalleled opportunities for comprehensive molecular profiling of thousands of individual cells, with expected major impacts across a broad range of biomedical research. The resulting datasets are often discussed using the term transcriptional landscape. However, the algorithmic analysis of cellular heterogeneity and patterns across such landscapes still faces fundamental challenges, for instance, in how to explain cell-to-cell variation. Current computational approaches attempt to achieve this usually in one of two ways [1]. Clustering assumes that data is composed of biologically distinct groups such as discrete cell types or states and labels these with a discrete variable—the cluster index. By contrast, inferring pseudotemporal orderings or trajectories of cells [2–4] assumes that data lie on a connected manifold and labels cells with a continuous variable—the distance along the manifold. While the former approach is the basis for most analyses of single-cell data, the latter enables a better interpretation of continuous phenotypes and processes such as development, dose response, and disease progression. Here, we unify both viewpoints.

A central example of dissecting heterogeneity in single-cell experiments concerns data that originate from complex cell differentiation processes. However, analyzing such data using pseudotemporal ordering [2, 5–9] faces the problem that biological processes are usually incompletely sampled. As a consequence, experimental data do not conform with a connected manifold and the modeling of data as a continuous tree structure, which is the basis for existing algorithms, has little meaning. This problem exists even in clustering-based algorithms for the inference of tree-like processes [10–12], which make the generally invalid assumption that clusters conform with a connected tree-like topology. Moreover, they rely on feature-space based inter-cluster distances, like the euclidean distance of cluster means. However, such distance measures quantify biological similarity of cells only at a local scale and are fraught with problems when used for larger-scale objects like clusters. Efforts for addressing the resulting high non-robustness of tree-fitting to distances between clusters [10] by sampling [11, 12] have only had limited success.

Partition-based graph abstraction (PAGA) resolves these fundamental problems by generating graph-like maps of cells that preserve both continuous and disconnected structure in data at multiple resolutions. The data-driven formulation of PAGA allows to robustly reconstruct branching gene expression changes across different datasets and, for the first time, enabled reconstructing the lineage relations of a whole adult animal [13]. Furthermore, we show that PAGA-initialized manifold learning algorithms converge faster, produce embeddings that are more faithful to the global topology of high-dimensional data, and introduce an entropy-based measure for quantifying such faithfulness. Finally, we show how PAGA abstracts transition graphs, for instance, from RNA velocity and compare to previous trajectory-inference algorithms. With this, PAGA provides a graph abstraction method [14] that is suitable for deriving interpretable abstractions of the noisy kNN-like graphs that are typically used to represent the manifolds arising in scRNA-seq data.

## Results

### PAGA maps discrete disconnected and continuous connected cell-to-cell variation

**Fig. 1:**
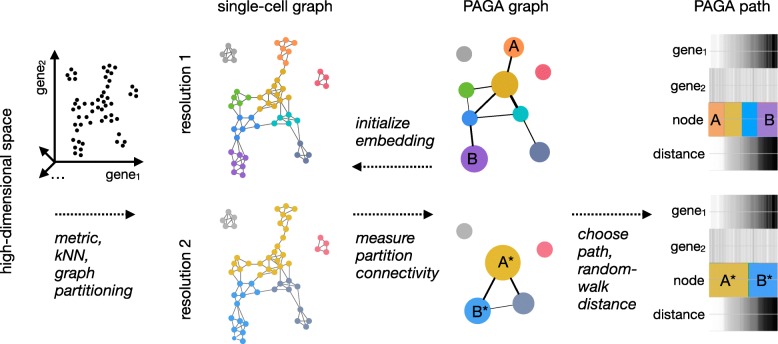
Partition-based graph abstraction generates a topology-preserving map of single cells. High-dimensional gene expression data is represented as a kNN graph by choosing a suitable low-dimensional representation and an associated distance metric for computing neighborhood relations—in most of the paper, we use PCA-based representations and Euclidean distance. The kNN graph is partitioned at a desired resolution where partitions represent groups of connected cells. For this, we usually use the Louvain algorithm, however, partitions can be obtained in any other way, too. A PAGA graph is obtained by associating a node with each partition and connecting each node by weighted edges that represent a statistical measure of connectivity between partitions, which we introduce in the present paper. By discarding spurious edges with low weights, PAGA graphs reveal the denoised topology of the data at a chosen resolution and reveal its connected and disconnected regions. Combining high-confidence paths in the PAGA graph with a random-walk-based distance measure on the single-cell graph, we order cells within each partition according to their distance from a root cell. A PAGA path then averages all single-cell paths that pass through the corresponding groups of cells. This allows to trace gene expression changes along complex trajectories at single-cell resolution

Both established manifold learning techniques and single-cell data analysis techniques represent data as a neighborhood graph of single cells *G*=(*V,E*), where each node in *V* corresponds to a cell and each edge in *E* represents a neighborhood relation (Fig. 1) [3, 15–17]. However, the complexity of *G* and noise-related spurious edges make it both hard to trace a putative biological process from progenitor cells to different fates and to decide whether groups of cells are in fact connected or disconnected. Moreover, tracing isolated paths of single cells to make statements about a biological process comes with too little statistical power to achieve an acceptable confidence level. Gaining power by averaging over distributions of single-cell paths is hampered by the difficulty of fitting realistic models for the distribution of these paths.

We address these problems by developing a statistical model for the connectivity of groups of cells, which we typically determine through graph-partitioning [17–19] or alternatively through clustering or experimental annotation. This allows us to generate a simpler PAGA graph *G*^∗^ (Fig. 1) whose nodes correspond to cell groups and whose edge weights quantify the connectivity between groups. Similar to modularity [20], the statistical model considers groups as connected if their number of inter-edges exceeds a fraction of the number of inter-edges expected under random assignment. The connection strength can be interpreted as confidence in the presence of an actual connection and allows discarding spurious, noise-related connections (Additional file 1: Note 1). While *G* represents the connectivity structure of the data at single-cell resolution, the PAGA graph *G*^∗^ represents the connectivity structure of the data at the chosen coarser resolution of the partitioning and allows to identify connected and disconnected regions of the data. Following paths along nodes in *G*^∗^ means following an ensemble of single-cell paths that pass through the corresponding cell groups in *G*. By averaging over such an ensemble of single-cell paths, it becomes possible to trace a putative biological process from a progenitor to fates in a way that is robust to spurious edges, provides statistical power, and is consistent with basic assumptions on a biological trajectory of cells (Additional file 1: Note 2). Note that by varying the resolution of the partitioning, PAGA generates graphs at multiple resolutions, which enables a hierarchical exploration of data (Fig. 1, Additional file 1: Note 1.3).

To trace gene dynamics at single-cell resolution, we extended existing random-walk-based distance measures (Additional file 1: Note 2, Reference [7]) to the realistic case that accounts for disconnected graphs. By following high-confidence paths in the abstracted graph *G*^∗^ and ordering cells within each group in the path according to their distance *d* from a progenitor cell, we trace gene changes at single-cell resolution (Fig. 1). Hence, PAGA covers both aspects of clustering and pseudotemporal ordering by providing a coordinate system (*G*^∗^,*d*) that allows us to explore variation in data while preserving its topology (Additional file 1: Note 1.6). PAGA can thus be viewed as an easily interpretable and robust way of performing topological data analysis [9, 21] (Additional file 1: Note 3).

### PAGA-initialized manifold learning produces topology-preserving single-cell embeddings

The computationally almost cost-free coarse-resolution embeddings of PAGA can be used to initialize established manifold learning and graph drawing algorithms like UMAP [22] and ForceAtlas2 (FA) [23]. This strategy is used to generate the single-cell embeddings throughout this paper. In contrast to the results of previous algorithms, PAGA-initialized single-cell embeddings are faithful to the global topology, which greatly improves their interpretability. To quantify this claim, we took a classification perspective on embedding algorithms and developed a cost function KL _geo_ (Box 1 and Additional file 1: Note 4), which captures faithfulness to global topology by incorporating geodesic distance along the representations of data manifolds in both the high-dimensional and the embedding space, respectively. Independent of this, PAGA-initialized manifold learning converges about six times faster with respect to established cost functions in manifold learning (Additional file 1: Figure S10)


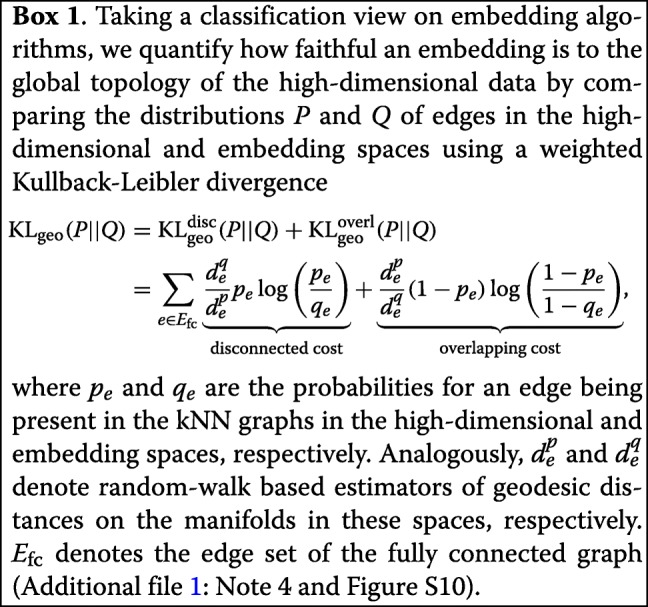


### PAGA consistently predicts developmental trajectories and gene expression changes in datasets related to hematopoiesis

Hematopoiesis represents one of the most extensively characterized systems involving stem cell differentiation towards multiple cell fates and hence provides an ideal scenario for applying PAGA to complex manifolds. We applied PAGA to simulated data (Additional file 1: Note 5) for this system and three experimental datasets: 2730 cells measured using MARS-seq [24], 1654 cells measured using Smart-seq2 [25], and 44,802 cells from a 10 × Genomics protocol [26]. These data cover the differentiation from stem cells towards cell fates including erythrocytes, megakaryocytes, neutrophils, monocytes, basophils, and lymphocytes.

**Fig. 2:**
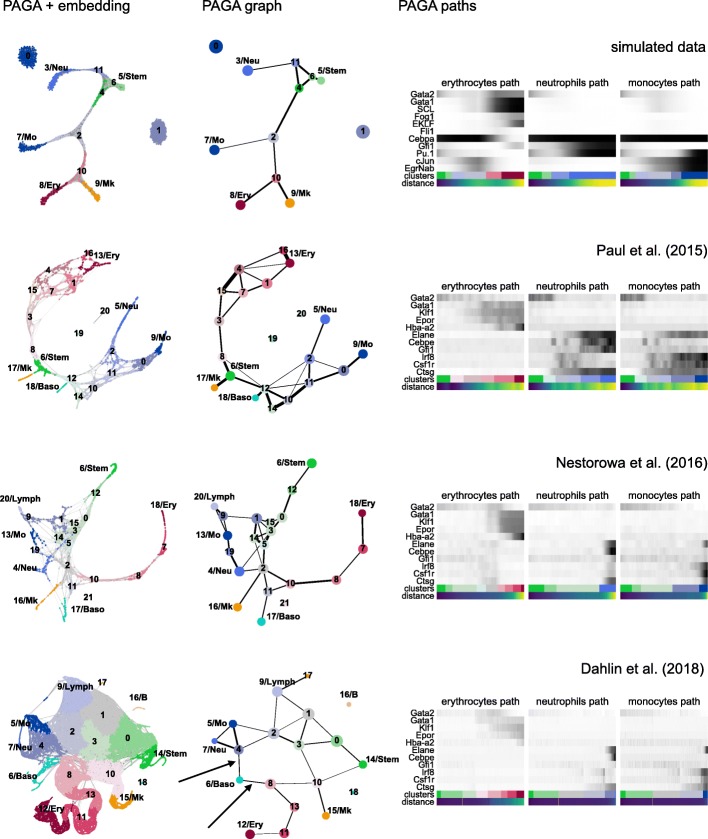
PAGA consistently predicts developmental trajectories and gene expression changes across datasets for hematopoiesis. The three columns correspond to PAGA-initialized single-cell embeddings, PAGA graphs, and gene changes along PAGA paths. The four rows of panels correspond to simulated data (Additional file 1: Note 5) and data from Paul et al. [24], Nestorowa et al. [25], and Dahlin et al. [26], respectively. The arrows in the last row mark the two trajectories to basophils. One observes both consistent topology of PAGA graphs and consistent gene expression changes along PAGA paths for 5 erythroid, 3 neutrophil, and 3 monocyte marker genes across all datasets. The cell type abbreviations are as follows: Stem for stem cells, Ery for erythrocytes, Mk for megakaryocytes, Neu for neutrophils, Mo for monocytes, Baso for basophils, B for B cells, Lymph for lymphocytes

The PAGA graphs (Fig. 2) capture known features of hematopoiesis, such as the proximity of megakaryocyte and erythroid progenitors and strong connections between monocyte and neutrophil progenitors. Under debate is the origin of basophils. Studies have suggested both that basophils originate from a basophil-neutrophil-monocyte progenitor or, more recently, from a shared erythroid-megakaryocyte-basophil progenitor [27, 28]. The PAGA graphs of the three experimental datasets highlight this ambiguity. While the dataset of Paul et al. falls in the former category, Nestorowa et al. falls in the latter and Dahlin et al., which has by far the highest cell numbers and the densest sampling, allows us to see both trajectories. Aside from this ambiguity that can be explained by insufficient sampling in Paul et al. and Nestorowa et al., even with the very different experimental protocols and vastly different cell numbers the PAGA graphs show consistent topology between the three datasets. Beyond consistent topology between cell subgroups, we find consistent continuous gene expression changes across all datasets—we observe changes of erythroid maturity marker genes (*Gata2*, *Gata1*, *Klf1*, *Epor*, and *Hba-a2*) along the erythroid trajectory through the PAGA graphs and observe sequential activation of these genes in agreement with known behavior. Activation of neutrophil markers (*Elane*, *Cepbe*, and *Gfi1*) and monocyte markers (*Irf8*, *Csf1r*, and *Ctsg*) are seen towards the end of the neutrophil and monocyte trajectories, respectively. While PAGA is able to capture the dynamic transcriptional processes underlying multi-lineage hematopoietic differentiation, previous algorithms often fail to robustly produce meaningful results (Additional file 1: Figures S8, S9, S10).

### PAGA maps single-cell data of whole animals at multiple resolutions

**Fig. 3:**
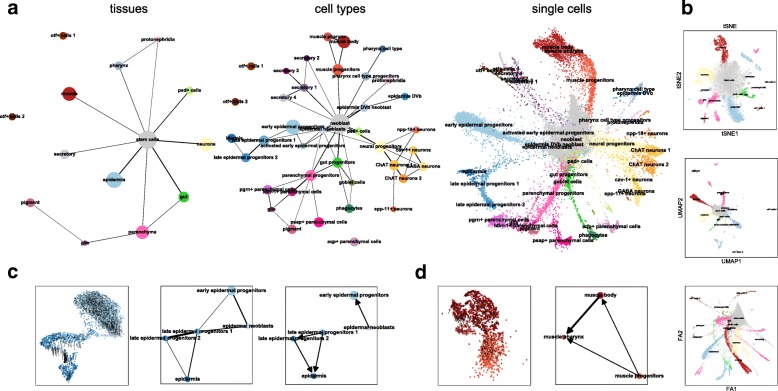
PAGA applied to a whole adult animal. **a** PAGA graphs for data for the flatworm *Schmidtea mediterranea* [13] at tissue, cell type, and single-cell resolution. We obtained a topologically meaningful embedding by initializing a single-cell embedding with the embedding of the cell-type PAGA graph. Note that the PAGA graph is the same as in Reference [13], only that here, we neither highlight a tree subgraph nor used the corresponding tree layout for visualization. **b** Established manifold learning for the same data violate the topological structure. **c**, **d** Predictions of RNA velocity evaluated with PAGA for two example lineages: epidermis and muscle. We show the RNA velocity arrows plotted on a single-cell embedding, the standard PAGA graph representing the topological information (only epidermis), and the PAGA graph representing the RNA velocity information

Recently, Plass et al. [13] reconstructed the first cellular lineage tree of a whole adult animal, the flatworm *Schmidtea mediterranea*, using PAGA on scRNA-seq data from 21,612 cells. While Plass et al. focussed on the tree-like subgraph that maximizes overall connectivity—the minimum spanning tree of *G*^∗^ weighted by inverse PAGA connectivity—here, we show how PAGA can be used to generate maps of data at multiple resolutions (Fig. 3a). Each map preserves the topology of data, in contrast to state-of-the-art manifold learning where connected tissue types appear as either disconnected or overlapping (Fig. 3b). PAGA’s multi-resolution capabilities directly address the typical practice of exploratory data analysis, in particular for single-cell data: data is typically reclustered in certain regions where a higher level of detail is required.

### PAGA abstracts information from RNA velocity

Even though the connections in PAGA graphs often correspond to actual biological trajectories, this is not always the case. This is a consequence of PAGA being applied to kNN graphs, which solely contain information about the topology of data. Recently, it has been suggested to also consider directed graphs that store information about cellular transition based on RNA velocity [29]. To include this additional information, which can add further evidence for actual biological transitions, we extend the undirected PAGA connectivity measure to such directed graphs (Additional file 1: Note 1.2) and use it to orient edges in PAGA graphs (Fig. 3c). Due the relatively sparsely sampled, high-dimensional feature space of scRNA-seq data, both fitting and interpreting an RNA velocity vector without including information about topology—connectivity of neighborhoods—is practically impossible. PAGA provides a natural way of abstracting both topological information and information about RNA velocity.

**Fig. 4:**
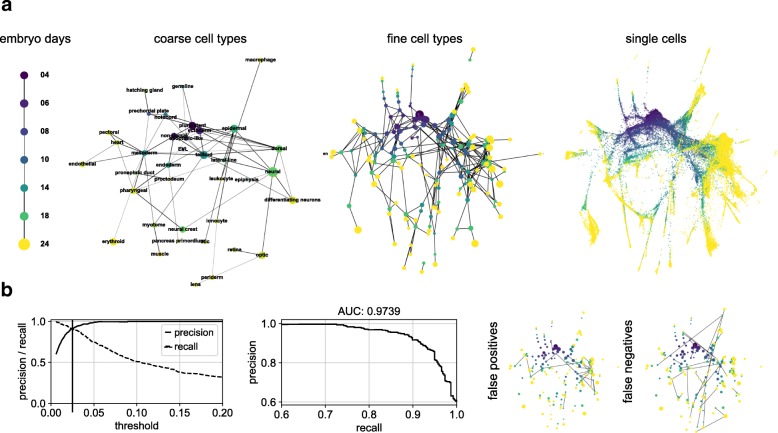
PAGA applied to zebrafish embryo data of Wagner et al. [30]. **a** PAGA graphs obtained after running PAGA on partitions corresponding to embryo days, coarse cell types, more fine-grained cell types, and a PAGA-initialized single-cell embedding. Cell type assignments are from the original publication. **b** Performance measurements of the PAGA prediction compared to the reference graph of Wagner et al. show high accuracy. False-positive edges and false-negative edges for the threshold indicated by a vertical line in the left panel are also shown

Next, we applied PAGA to 53,181 cells collected at different developmental time points (embryo days) from the zebrafish embryo [30]. The PAGA graph for partitions corresponding to embryo days accurately recovers the chain topology of temporal progression, whereas the PAGA graph for cell types provides easily interpretable overviews of the lineage relations (Fig. 4a). Initializing a ForceAtlas2 layout with PAGA coordinates from fine cell types automatically produced a corresponding, interpretable single-cell embedding (Fig. 4a). Wagner et al. [30] both applied an independently developed computational approach with similarities to PAGA (Additional file 1: Note 3) to produce a coarse-grained graph and experimentally validated inferred lineage relations. Comparing the PAGA graph for the fine cell types to the coarse-grained graph of Wagner et al. reproduced their result with high accuracy (Fig. 4b).

### PAGA increases computational efficiency and interpretability in general exploratory data analysis and manifold learning

Comparing the runtimes of PAGA with the state-of-the-art UMAP [22] for 1.3 million neuronal cells of 10 × Genomics [31] we find a speedup of about 130, which enables interactive analysis of very large-scale data (90 s versus 191 min on 3 cores of a small server, tSNE takes about 10 h). For complex and large data, the PAGA graph generally provides a more easily interpretable visualization of the clustering step in exploratory data analysis, where the limitations of two-dimensional representations become apparent (Additional file 1: Figure S12). PAGA graph visualizations can be colored by gene expression and covariates from annotation (Additional file 1: Figure S13) just as any conventional embedding method.

### PAGA is robust and qualitatively outperforms previous lineage reconstruction algorithms

To assess how robustly graph and tree-inference algorithms recover a given topology, we developed a measure for comparing the topologies of two graphs by comparing the sets of possible paths on them (Additional file 1: Note 1.4, Figure S4). Sampling widely varying parameters, which leads to widely varying clusterings, we find that the inferred abstraction of topology of data within the PAGA graph is much more robust than the underlying graph clustering algorithm (Additional file 1: Figure S5). While graph clustering alone is, as any clustering method, an ill-posed problem in the sense that many highly degenerate quasi-optimal clusterings exist and some knowledge about the scale of clusters is required, PAGA is not affected by this.

Several algorithms [5, 10–12] have been proposed for reconstructing lineage trees (Additional file 1: Note 3, [4]). The main caveat of these algorithms is that they, unlike PAGA, try to explain any variation in the data with a tree-like topology. In particular, any disconnected distribution of clusters is interpreted as originating from a tree. This produces qualitatively wrong results already for simple simulated data (Supplementary Figure 6) and only works well for data that clearly conforms with a tree-like manifold (Supplementary Figure 7). To establish a fair comparison on real data with the recent popular algorithm, Monocle 2, we reinvestigated the main example of Qiu et al. [5] for a complex differentiation tree. This example is based on the data of Paul et al. [24] (Fig. 2), but with cluster 19 removed. While PAGA identifies the cluster as disconnected with a result that is unaffected by its presence, the prediction of Monocle 2 changes qualitatively if the cluster is taken into account (Supplementary Figure 8). The example illustrates the general point that real data almost always consists of dense and sparse—connected and disconnected—regions, some tree-like, some with more complex topology.

## Conclusions

In view of an increasing number of large datasets and analyses for even larger merged datasets, PAGA fundamentally addresses the need for scalable and interpretable maps of high-dimensional data. In the context of the Human Cell Atlas [32] and comparable databases, methods for their hierarchical, multi-resolution exploration will be pivotal in order to provide interpretable accessibility to users. PAGA allows to present information about clusters or cell types in an unbiased, data-driven coordinate system by representing these in PAGA graphs. In the context of the recent advances of the study of simple biological processes that involve a single branching [6, 7], PAGA provides a similarly robust framework for arbitrarily complex topologies. In view of the fundamental challenges of single-cell resolution studies due to technical noise, transcriptional stochasticity, and computational burden, PAGA provides a general framework for extending studies of the relations among single cells to relations among noise-reduced and computationally tractable groups of cells. This could facilitate obtaining clearer pictures of underlying biology.

In closing, we note that PAGA not only works for scRNA-seq based on distance metrics that arise from a sequence of chosen preprocessing steps, but can also be applied to any learned distance metric. To illustrate this point, we used PAGA for single-cell imaging data when applied on the basis of a deep-learning-based distance metric. Eulenberg et al. [33] showed that a deep learning model can generate a feature space in which distances reflect the continuous progression of cell cycle. Using this, PAGA correctly identifies the biological trajectory through the interphases of cell cycle while ignoring a cluster of damaged and dead cells (Additional file 1: Figure S14).

## Methods

### Preprocessing scRNA-seq data

We preprocess scRNA-seq data as commonly done following steps mostly inspired by Seurat [34] in the implementation of Scanpy [35]. These steps consist in basic filtering of the data, total count normalization, log1p logarithmization, extraction of highly variable genes, a potential regression of confounding factors, and a scaling to *z*-scores. On this corrected and homogenized representation of the count data, we perform a PCA and represent the data within the reduced space of principal components. As an alternative to this “classical” procedure, which is built on the PCA representation of the data, one might consider using the latent space representation of neural network model such as scVI for scRNA-seq data [36], or as the classifier discussed in Additional file 1: Note 5.6. Detailed parameters used for the processing can be found in Additional file 1: Note 5 and at https://github.com/theislab/paga. In the GitHub repository, each figure of the paper is reproduced in a dedicated notebook.

### Graph construction

Using the compressed and denoised representation of the data in the previous step, we construct a symmetrized kNN-like graph, typically using the approximate nearest neighbor search within UMAP [22]. While one might potentially choose different distance metrics, we always choose Euclidean distance. Depending on user choice, the graph is either weighed using adaptive Gaussian kernels [7] or the exponential kernel within UMAP [22]. For all results shown in the manuscript, we used the exponential kernel.

### Graph partitioning and abstraction

We consider all partitionings of interest of the kNN-like graph. To determine those, typically, we use the Louvain algorithm in the implementation of [37] at suitable resolutions, but PAGA works with any underlying clustering algorithm or experimentally generated groupings of observations. In the present work, we exclusively used the Louvain algorithm.

In the conventional undirected case, for each partitioning, we generate a PAGA graph using the “PAGA connectivity measure” defined in Additional file 1: Eq. (11). This measure is a test statistic quantifying the degree of connectivity of two partitions and has a close relation with modularity [20]. For each pair of clusters, PAGA connectivity is the ratio of the number of inter-edges between the clusters normalized with the number of inter-edges expected under random assignment of edges.

In the directed case, in which we typically abstract a “velocity graph” originating from RNA velocity [29], we consider the ratio of arrows Additional file 1: Eq. (14), which are in- and outgoing for each pair of partitions to quantify a tendency of transition between partitions.

### Pseudotime estimation

For estimating pseudotime, we use an extended version of diffusion pseudotime (DPT) Reference [7] that accounts for disconnected graphs. The extension consists in a simple modification of the original algorithm that accounts for disconnected Eigen-subspaces of the graph adjacency matrix, which results in multiple subspaces of Eigen value 1 of the graph transition matrix. Practically, we assign an infinite distance to cells that reside in disconnected clusters and compute distances among cells within connected regions in the graph as it would be done in DPT. See Additional file 1: Note 2, both for details and for a review of random-walk-based distances. For instance, we show the close relation of DPT to mean commute distance.

### Consistent embeddings across resolutions

PAGA achieves consistent (i.e., minimally displaced in the embedding space) and topology-preserving embeddings by initializing an embedding of a fine-grained graph using the coordinates of a coarse-grained graph. For this initialization, the positions of nodes of the fine-grained graph that belong to a group corresponding to a node in the coarse-grained graph are randomly distributed in a non-overlapping rectangular region around the position of that node. This procedure is repeated for all nodes of the coarse-grained graph. Non-overlapping regions are trivially ensured by choosing rectangles with half-edge lengths of half the distance to the nearest neighbor in the coarse-grained embedding.

Conversely, for a given fine-grained graph, we position nodes in the coarse-grained graph by placing them on the median coordinates of the positions of the corresponding nodes in the fine-grained graph.

## Additional file

Additional file 1 Supplementary figures and notes. (PDF 6324 kb)
