# Supplementary material for: PAGA: graph abstraction reconciles clustering with trajectory inference through a topology preserving map of single cells
Source: Genome Biol. 2019 Mar 19;20:59. doi: 10.1186/s13059-019-1663-x (PMC6425583; doi:10.1186/s13059-019-1663-x)
Supplement: Supplementary file 1 — Supplementary figures and notes. (PDF 6324 kb) [file 13059_2019_1663_MOESM1_ESM.pdf]

Supplemental Figures

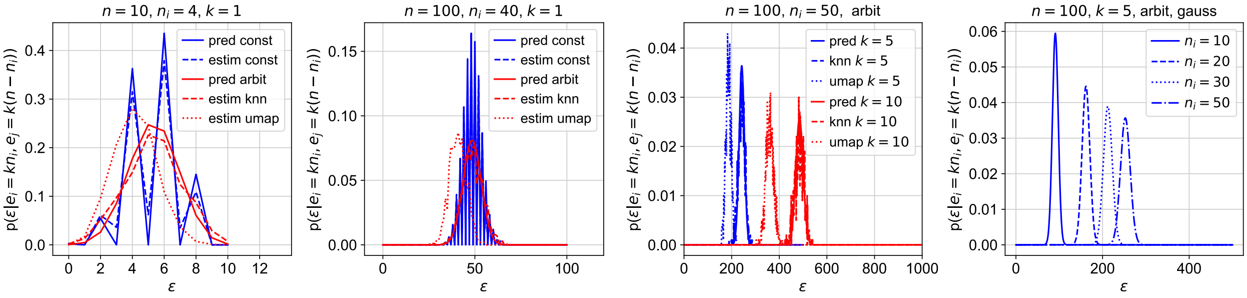

Figure S1 | Distribution of the number of inter edges for random bipartitions of fixed degree and for empirical knn graphs. This distribution serves as null distribution, see Figure S2. Graphs with n nodes and degree k are sampled. They are randomly bipartitioned, where the size of one bipartition is n_i and the remainder of the graph gives raise to a second partition of size $n - n_i$. Shown are both sampling-based estimates and model predictions for different parameters.

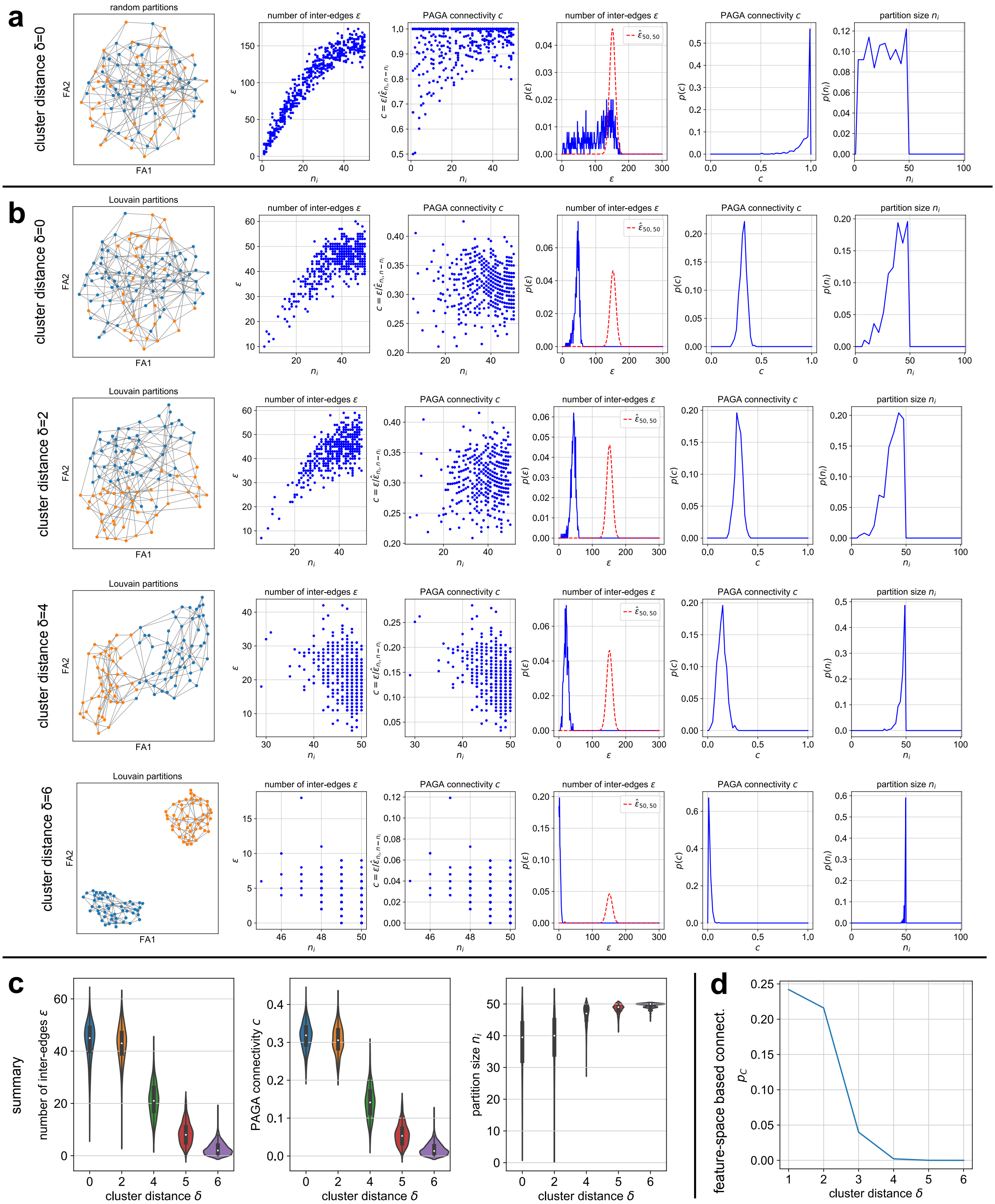

Figure S2 | The PAGA connectivity measure provides a low-variance estimate of inter-partition connectivity that is approximately independent of partition sizes. While in the first three rows (**a**, **b**), the number of inter-partition edges ε (second column) varies as a parabola with partition size n_i, the PAGA connectivity (third column) is essentially independent of n_i. The first column shows an embedding of the partitioned graph, the second column shows the number of inter-edges as a function of partition size, the third column shows PAGA connectivity (11) as a function of partition size, the forth column shows the distribution of inter-edges, the fifth column the distribution of PAGA connectivity and the sixth column the distribution of partition sizes. Data has been sampled from the Gaussian mixture model (13) for different cluster distances δ. For each value of δ (each row in the figure), we sample 100 observations $\boldsymbol{x}_\iota$ in 500 simulations. **a,** Results for random bipartitions. **b,** Results for Louvain bipartitions. **c**, Summary of **b**. The box plots within the violins show the inner quartiles. **d,** Feature-space based estimate of connectivity (20).

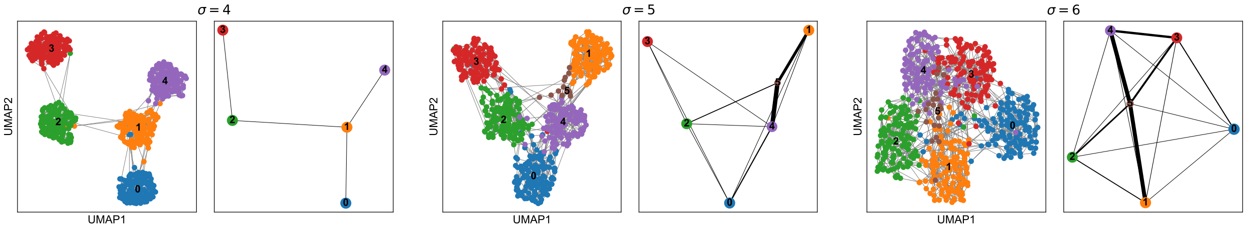

Figure S3 | Connectivity of clusters sampled from Gaussian mixture model for different values of the standard deviation. Here, we show samples from three Gaussian mixture models, which display different degrees of clustering structure: the number of centers is fixed to 5 but the standard deviation σ is increases from 4 to 6. The figure makes evident that the same number of inter-edges for a small cluster leads to higher confidence in a connection than for a large cluster.

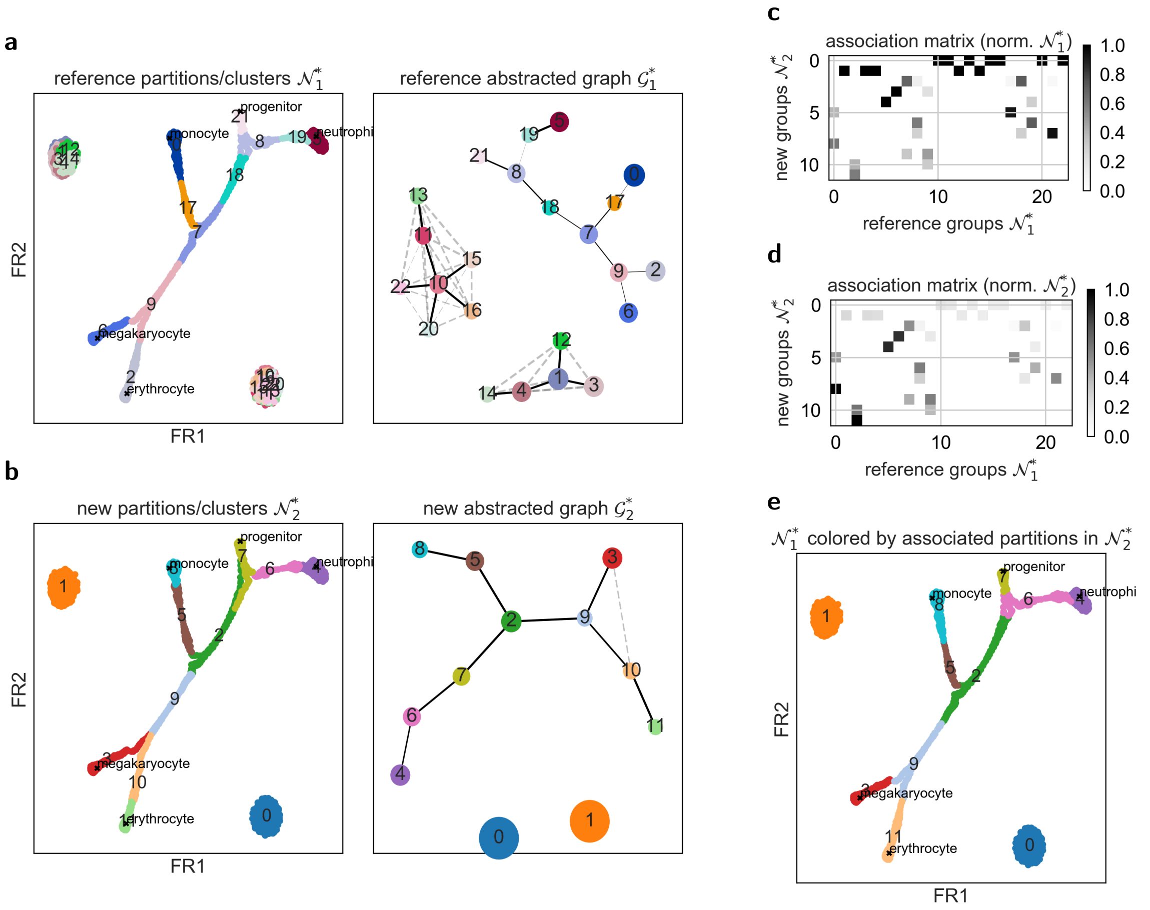

Figure S4 | Illustration of multi-resolution analysis for simulated data. Note that unlike in Figures 1, 2 and 3 of the main text and several other supplemental figures, here, we did *not* enforce consistency between graph layouts across resolutions. **a, b,** Partitions obtained using Louvain clustering in two runs with different parameters, equivalent to those shown in Figure 2a: both abstracted graphs describe the same topology. Note that in Figure 2a. **c, d,** Map between clusters of different resolutions. **e,** Reference partitions colored with the associated new partition that has the largest overlap.

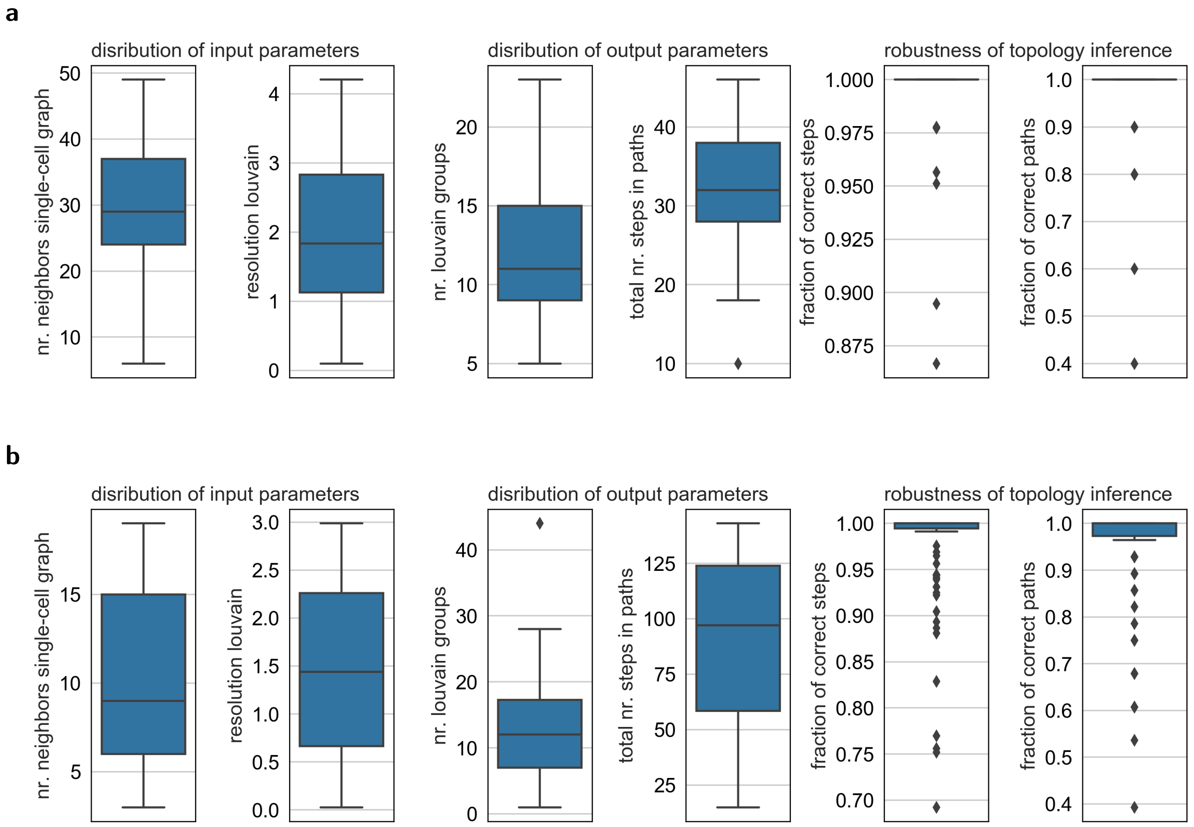

Figure S5 | Robustness of PAGA. Sampling a wide variety of the input parameters (numbers of neighbors in the kNN graph and resolution of the Louvain partitioning) results in vastly varying numbers of partitions, hence vastly different clusterings of the data; note the large spread of the number of Louvain partitions. Nonetheless, the topology is robustly inferred. **a,** Simulated data as in Figure 2. **b,** Data of Reference [1] as in Figure 2.

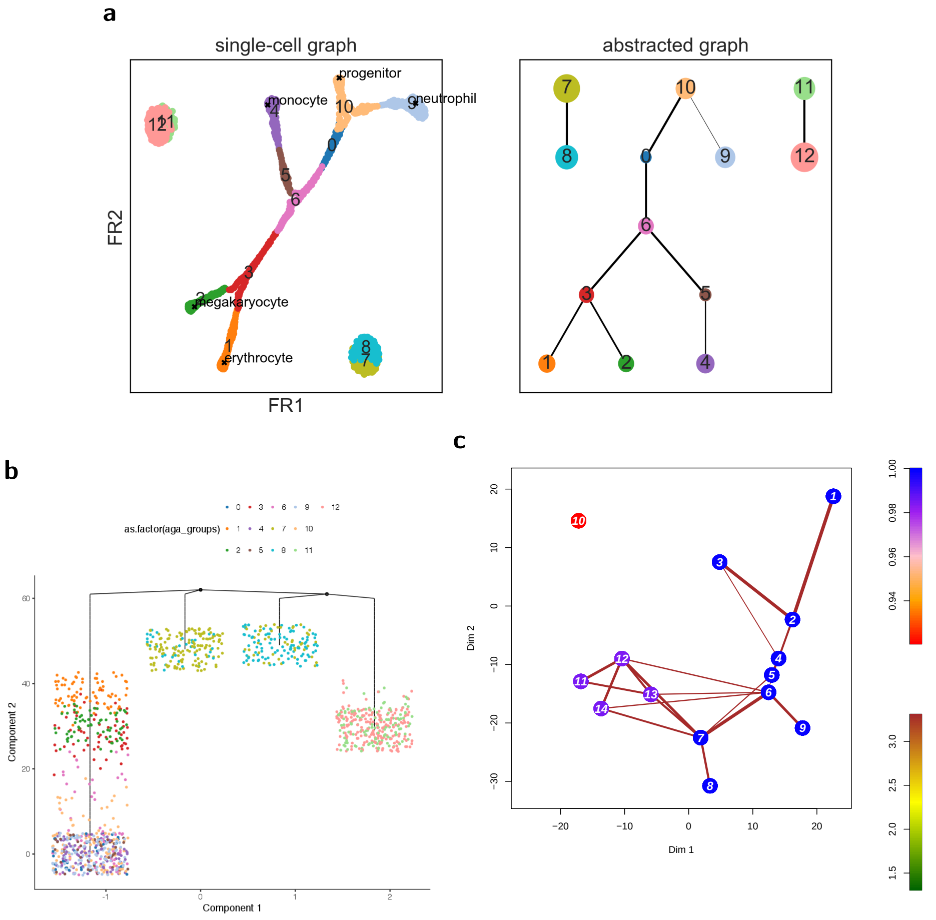

Figure S6 | Comparison with Monocle 2 and stemID 2 for simulated myeloid differentiation and clusters. a, Prediction of graph abstraction, analogous to Figure 2. **b,** Prediction of Monocle 2 [2], the best result after testing several parameters for the latent-space dimension. The clusters (groups 7, 8 and 11, 12 in panel a) dictate the shape of the inferred tree, being responsible for three of the four observed branches. The continuous manifold is not resolved at all. The same coloring as in panel a is used. **c,** Prediction of the lineage tree of stemID 2, the successor of stemID [3]. The author of stemID, D. Grün, ran the simulation himself. The coloring and numbering of groups is chosen internally by stemID 2.

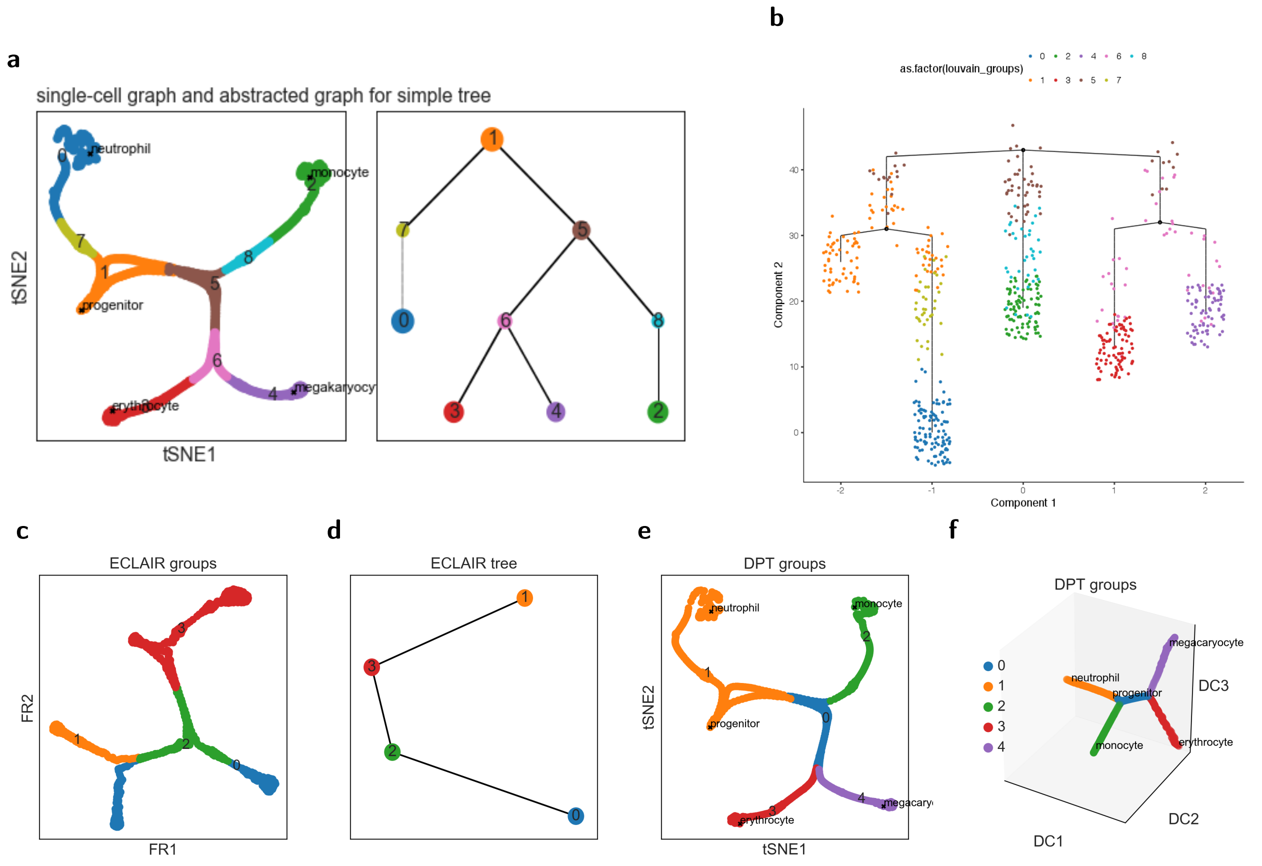

Figure S7 | Comparisons for simulated myeloid differentiation giving rise to a simple tree-like manifold. Results using, **a,** graph abstraction, **b,** Monocle 2 [2], **c, d,** ECLAIR [4] and **e, f,** DPT [5] in its hierarchical implementation [6].

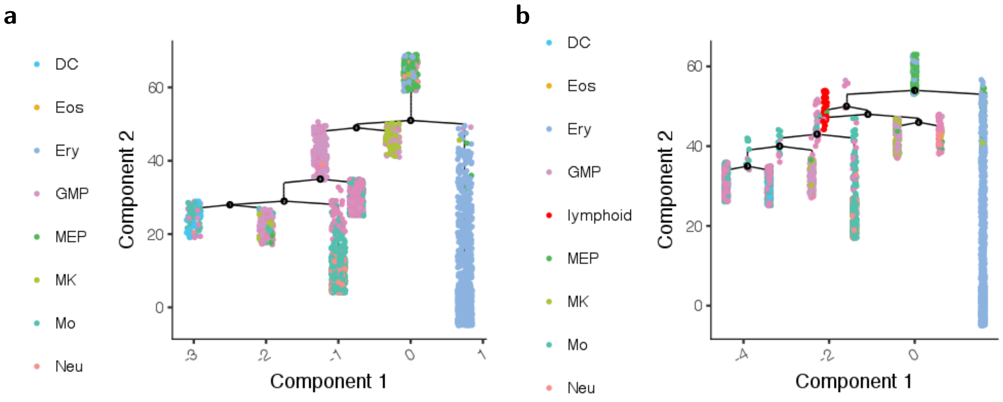

Figure S8 | Monocle 2 for data of Paul *et al.* [1]. **a,** Monocle 2's multiple branching example of Supplemental Figure 16 of Reference [2] using the same color coding as in the original publication. **b,** Rerunning Monocle 2 with the exact same parameters as for panel b, but keeping the lymphoids as in Figure 2a. The resulting tree changed dramatically and is no longer biologically meaningful. For example, the lymphoid cells are placed in the myeloid differentiation and myeloid progenitors (GMP) and monocytes (Mo) are distributed over all terminal states. As Monocle 2 does not provide confidence measures, the user erroneously expects all results to predicted with high confidence.

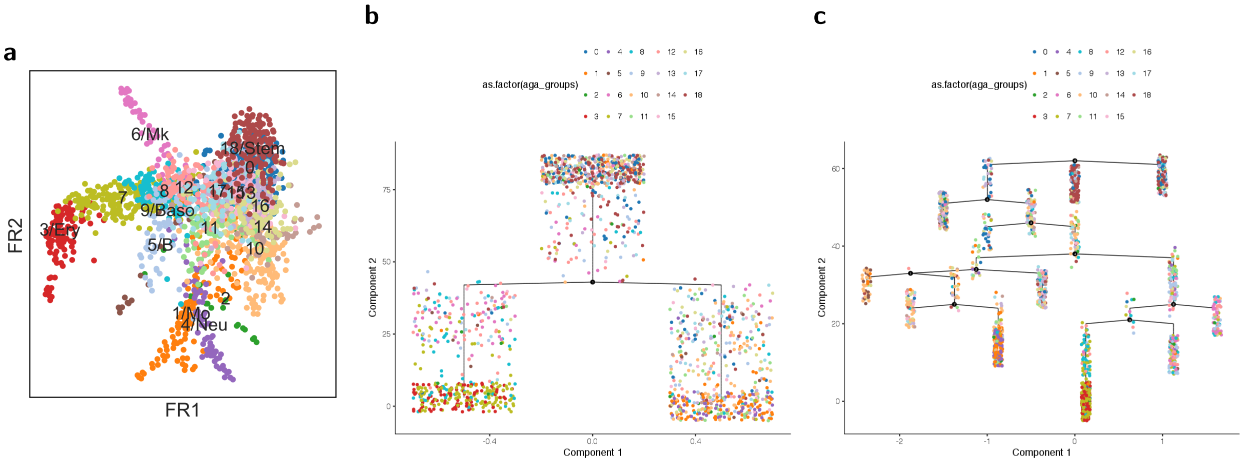

Figure S9 | Comparison with Monocle 2 for data of Nestorowa *et al.* [7]. **a,** FR embedding colored by cell type annotation. This is an alternative embedding to the one shown in Figure 2 based on the graph that was not denoised. **b,** Running Monocle 2 for a latent space dimension of 4 underestimates the complexity of the differentiation manifold. **c,** Running Monocle 2 for a latent space dimension of 10 recovers the expected biology that late erythrocytes (3/Ery) and megakaryoctes (6/Mk) appear in the same region of the tree. Nonetheless, there are qualitative inconsistencies: neutrophils (4/Neu) and monocytes (1/Mo) appear in the same terminal branch. Megakaryocytes (6/Mk) appear in two branches. Basophils (9/Baso) appear as progenitors of erythrocytes and megakaryoctes and the disconnected B cells appear within the tree.

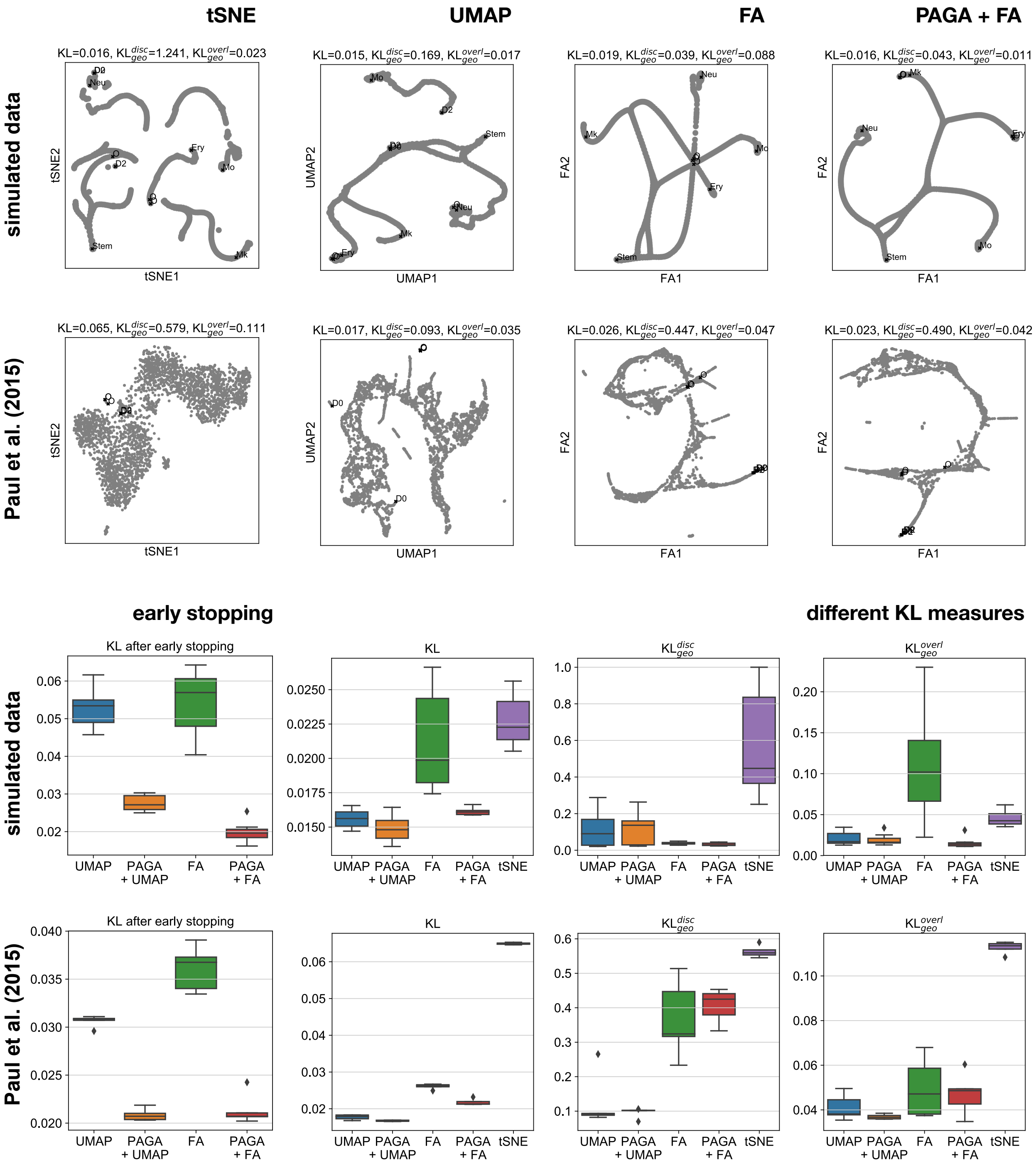

Figure S10 | Performance of different embedding algorithms in representing local and global topological properties of high-dimensional data. Using PAGA as an initialization for established manifold learning algorithms both leads to the best quality embeddings and enables early stopping. See (43) for the definition of the conventional KL divergence, which quantifies the preservation of local topology, and (46) for geodesic KL divergence $\mathrm{KL}_{\mathrm{geo}}$, which also accounts for preservation of global topology. Highlighted are points in the embedding that lead to strong violations of global topology (overlapping "O" and disconnected "D" points).

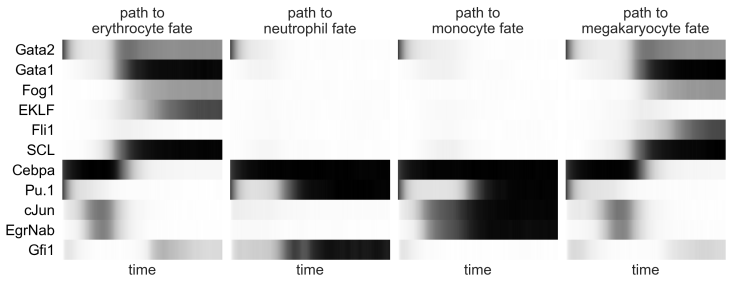

Figure S11 | Simulated data for myeloid differentiation. Four representative realizations of time series representing differentiation to four different fates. These time series have been sampled from (47) after transformation to a system of stochastic differential equations Reference [6, 8].

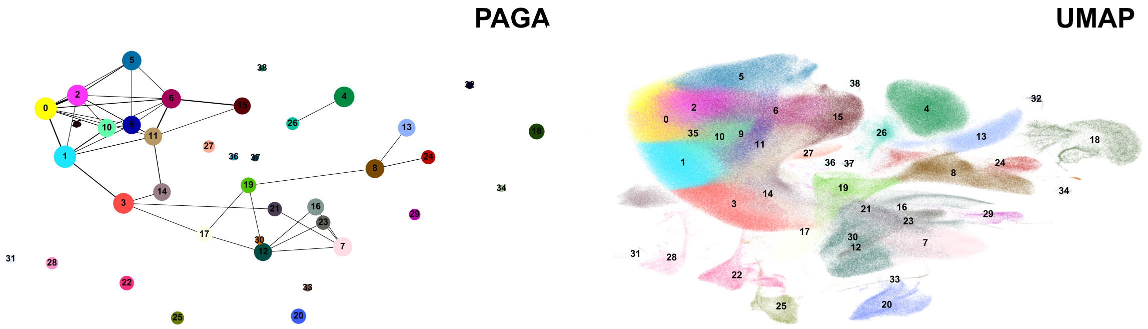

Figure S12 | PAGA and UMAP on 1.3 million neurons dataset from 10x Genomics [9]. While PAGA takes about 90 s of computation time, UMAP takes about 3 h. Due to overlaying groups, UMAP blurs the topological structure and visually suggests too much connectivity — it suggests connections where there actually are none, as shown by PAGA. Consider the example of cluster 19, which UMAP suggests to connect to cluster 21 whereas it actually connects to clusters 17, 30 and 8. Note that this figure is the only instance of the main text in which we used the default initialization of UMAP and not the PAGA coordinates.

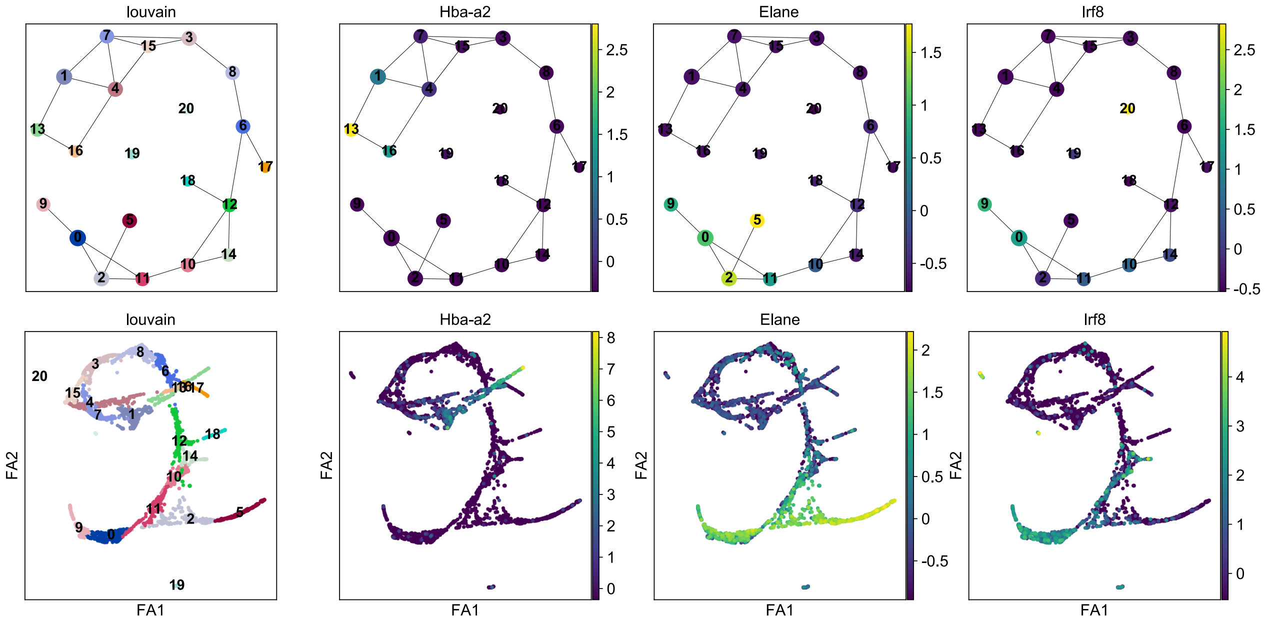

Figure S13 | Annotation of Louvain clusters for hematopoietic data of Paul *et al.* using PAGA and ForceAtlas2. PAGA and the ForceAtlas2 (FA) embedding were computed with default parameters. In contrast to the PAGA-initialized embedding of Figure 2, the single-cell layout shows overlapping structure. While this is a relatively small and simple dataset an interpretable single-cell embedding, it exemplifies that the PAGA graph can be used as an even easier accessible visualization of the data. Both PAGA and single-cell graph show the Louvain clusters, an erythroid branch marked by *Hba-a2*, a neutrophil branch marked by *Elane* and a monocyte branch marked by *Irf8*.

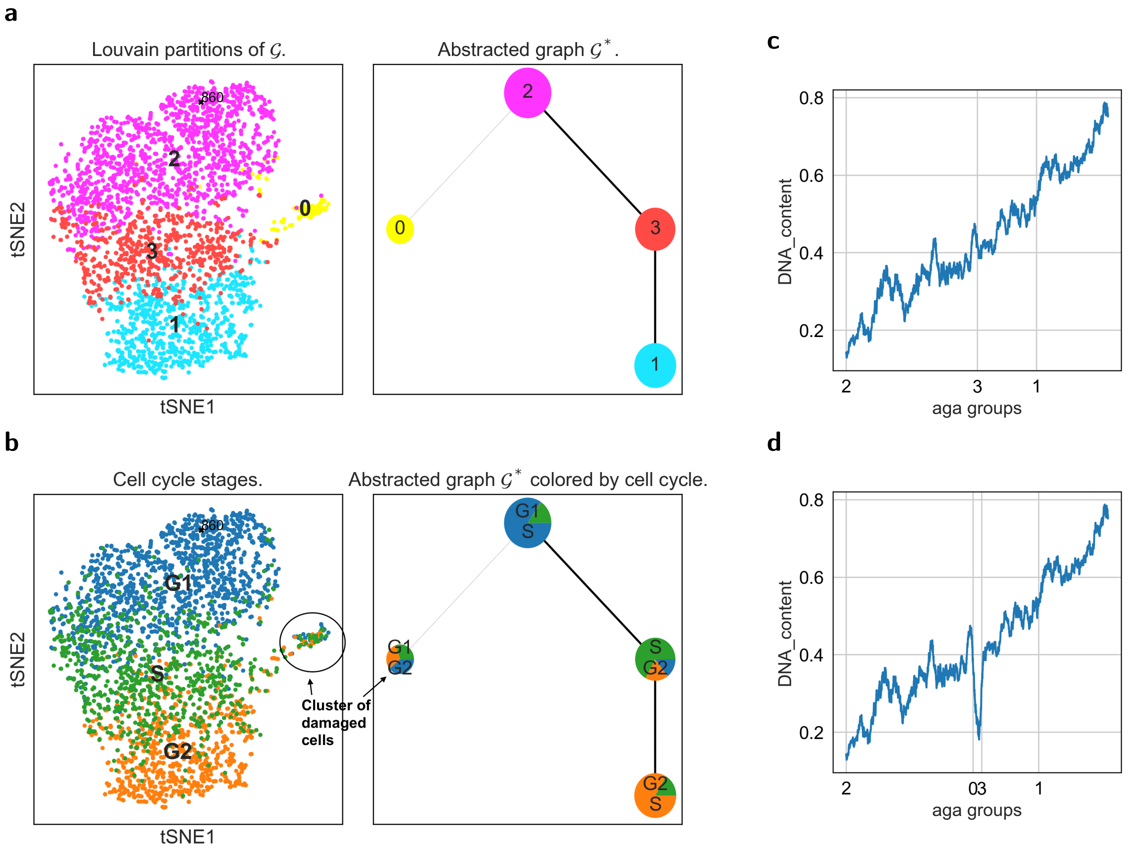

Figure S14 | Abstracted graph for deep learning based feature space. Analyzing single-cell images via a deep learning based distance metric. Graph abstraction correctly recognizes the cluster of damaged cells as not belonging to the biological path that corresponds to cell cycle evolution through the interphases G1, S and G2. **a,** Abstracted graph with Louvain partitions. **b,** Associated cell cycle phases. **c,** DNA content along a valid path in the abstracted graph. **d,** The DNA content along an invalid path that involves the damaged cells shows a clear non-biological kink.

Supplemental Notes

Contents

1 Theoretical background of PAGA **12**

1.1 PAGA for mapping connectivity between partitions . . . 13

1.1.1 Graphs with constant degree distribution . . . 13

1.1.2 Graphs with arbitrary degree distribution . . . 13

1.1.3 Comparing model predictions with sampling based estimates for kNN graphs 14

1.1.4 Statistical test for disconnectedness and PAGA connectivity measure . . . 15

1.1.5 Relation to modularity . . . 15

1.1.6 Feature-space based connectivity . . . 16

1.1.7 Benchmarks for Louvain-partitioned kNN graphs for clustering data . . . 17

1.2 PAGA for mapping transitions between partitions . . . 18

1.3 PAGA for multi-resolution analysis of data . . . 19

1.4 Robustness of PAGA . . . 20

1.5 Remarks on generating and partitioning single-cell graphs . . . 21

1.6 Remarks on the reconciliation of clustering with trajectory inference algorithms . . . 22

2 Random walks on graphs **22**

3 Comparisons with previous approaches **25**

3.1 Conceptual comparisons . . . 25

3.2 Simulated minimal examples with known ground truth . . . 27

3.3 Hematopoiesis . . . 27

3.4 Runtimes . . . 27

4 Faithfulness of embeddings to global topology **28**

5 Datasets **30**

5.1 Simulated dataset for hematopoiesis . . . 30

5.2 One million neurons . . . 31

5.3 Experimental datasets for hematopoiesis . . . 31

5.4 Planaria . . . 31

5.5 Zebrafish embryo . . . 31

5.6 Deep-learning-processed image data . . . 31

Supplemental Note 1: Theoretical background of PAGA

The simplest measure for connectivity of two partitions of G is the number of connecting edges between two partitions. However, this number alone depends strongly on the partition sizes, which prevents its meaningful interpretation. Instead, we compute a statistic of this number that measures confidence in an actual connection between two partitions of G, as opposed to a connection that is based on spurious edges. Hence, in the visualization of PAGA graphs, edge width should be interpreted as a measure of connectivity whose strength indicates the presence of an actual connection. The present note is quite long, you can jump to subsection 1.1.7 if you are only interested in numerical benchmark results and a summary figure.

Note that topological data analysis (TDA) [10] uses clustering algorithms that lead to overlapping clusters and by that circumvents a statistical definition of a connectivity measure: two clusters are connected if they have finite overlap. In contrast to the easily interpretable, essentially parameter-free and computational efficient Louvain algorithm for modularity optimization [11] — which therefore has become the standard for single-cell analysis [12] — TDA comes with problems in all three

respects and is hence not widely used despite it's recent proposition for scRNA-seq [13]. Also, to date, overlapping graph partitioning algorithms, which also provide a notion of connectivity based on overlaps are, to date, no practical alternative.

Supplemental Note 1.1: PAGA for mapping connectivity between partitions

In order to derive a statistical model for connectivity between partitions of kNN graphs of data we need to study the distribution of inter-edges between partitions of the graph. In the simplest case, the model is a distribution conditioned on partition sizes. However, a priori, it is somewhat unclear on which degree distribution of a graph a model for inter-edges should be based. By construction, kNN graphs display a degree distribution "close to a constant degree k". However, this is not an exact constraint and can be violated. As model systems, we investigate graphs with constant and arbitrary degree distribution. We will then choose the appropriate model by comparing model predictions with the empirical estimates of inter-edge distributions that we obtain from kNN graphs.

1.1.1 Graphs with constant degree distribution

Consider the case of a partitioned directed graph $G = (V, E)$ with h_i half-edges or "edge stubs" attached to nodes in a given partition i, h_j half-edges attached to nodes in a partition j and a total of $h = \sum_{i'} h_{i'}$ half-edges, which we require to be even. Connected among each other, the half-edges give rise to the $e = \frac{h}{2} = |E|$ edges of G. Assuming half-edges to be distinguishable and randomly connecting half-edges constrained to a constant outdegree distribution of $k = 1$ in i and indegree distribution of $k = 1$ in j, one has $\Omega^{\text{total}}_{\text{const}}$ possibilities of combining edges and $\Omega^{\varepsilon}_{\text{const}}$ possibilities so that exactly ε_{ij} inter-edges from partitions i and j are obtained, with

$$\Omega^{\text{const}}_{\text{total}|h} = \frac{(h)!}{2^{\frac{h}{2}}(\frac{h}{2})!}, \quad \Omega^{\text{const}}_{\varepsilon_{ij}|h_i,h_j,h} = \frac{h_i!h_j!}{\varepsilon_{ij}!2^{(h_i-\varepsilon_{ij})/2}(\frac{h_i-\varepsilon_{ij}}{2})!2^{(h_j-\varepsilon_{ij})/2}(\frac{h_j-\varepsilon_{ij}}{2})!}\frac{(h-h_i-h_j)!}{2^{\frac{h-h_i-h_j}{2}}(\frac{h-h_i-h_j}{2})!}. \tag{1}$$

The resulting probability is $p^{\text{directed}}_{\text{const}}(\varepsilon_{ij}|h_i, h_j, h) = \Omega^{\text{const}}_{\varepsilon_{ij},h_i,h_j,h}/\Omega^{\text{const}}_{\text{total},h}$.[1] We now wish an estimate for the number of inter-edges that is useful also for undirected graphs, hence, the sampling procedure should be symmetric between i and j and the distribution of interest is the one of the summed inter-edges $\varepsilon = \varepsilon_{ij} + \varepsilon_{ji}$ when connecting outgoing edges both from i and j,

$$p_{\text{const}}(\varepsilon|h_i, h_j, h) = \sum_{\varepsilon_{ij}=0}^{\varepsilon} p^{\text{directed}}_{\text{const}}(\varepsilon_{ij}|h_i, h_j, h)p^{\text{directed}}_{\text{const}}(\varepsilon - \varepsilon_{ij}|h_j, h_i, h). \tag{2}$$

1.1.2 Graphs with arbitrary degree distribution

Consider again a partitioned directed graph $G = (V, E)$, but now with $e = |E|$ edges and $n = |V|$ nodes. Imagine we have e_i dangling outgoing edges attached to n_i nodes in partition i and we randomly connect each of the dangling edges to a random node in the graph. Enumeration as before gives $\Omega^{\text{arbit}}_{\text{total}}$ possibilities of connecting these edges and $\Omega^{\text{arbit}}_{\varepsilon_{ij}}$ possibilities so that exactly ε_{ij} inter-edges from partition i to j are obtained,

$$\Omega^{\text{arbit}}_{\text{total}|e_i,n} = (n-1)^{e_i}, \quad \Omega^{\text{arbit}}_{\varepsilon_{ij}|e_i,n_j,n} = \binom{e_i}{\varepsilon_{ij}} n_j^{\varepsilon_{ij}}(n-n_j-1)^{e_i-\varepsilon_{ij}}, \tag{3}$$

where $\Omega^{\text{arbit}}_{\varepsilon_{ij}|e_i,n_j,n}$ is the product of the possibilities for ε_{ij} inter-edges from i to j and the total possibilities of edges among the remaining nodes i. Upon definition of $\theta_i = \frac{n_i}{n-1}$, the resulting

[1] This is a simple expression in the bipartitioned case $h = h_i + h_j$

$$p^{\text{directed}}_{\text{const}}(\varepsilon_{ij}|h_i, h_j, h = h_i + h_j) = 2^{\varepsilon_{ij}}\binom{(h_i+h_j)/2}{\varepsilon_{ij}}\binom{(h_i+h_j)/2-\varepsilon_{ij}}{(h_i-\varepsilon_{ij})/2}.$$

probability becomes a binomial

$$p_{\text{arbit}}^{\text{directed}}(\varepsilon_{ij}|e_i, n_i, n_j) = \binom{e_i}{\varepsilon_{ij}} \theta_j^{\varepsilon_{ij}} (1-\theta_j)^{e_i - \varepsilon_{ij}}, \tag{4}$$

or equivalently,

$$\varepsilon_{ij} \sim \text{Binomial}(e_i, \theta_j), \quad \text{E}[\varepsilon_{ij}] = e_i\theta_j = \frac{e_i n_j}{n-1}, \quad \text{Var}[\varepsilon_{ij}] = e_i\theta_j(1-\theta_j) = \frac{e_i n_j(n-n_j-1)}{n-1}. \tag{5}$$

We can interpret ε_{ij} as the number of "hitting" partition j when randomly distributing the e_i edges of partition i where θ_j is the probability for "hitting" j for a single edge from i.

As before, we wish the sampling procedure to be symmetric between i and j, hence are interested in the distribution of $\varepsilon = \varepsilon_{ij} + \varepsilon_{ji}$, which reads

$$p_{\text{arbit}}(\varepsilon|e_i, e_j, n_i, n_j, n) = \sum_{\varepsilon_{ij}=0}^{\varepsilon} \binom{e_i}{\varepsilon_{ij}} \binom{e_j}{\varepsilon - \varepsilon_{ij}} \theta_j^{\varepsilon_{ij}} (1-\theta_j)^{e_i-\varepsilon_{ij}} \theta_i^{\varepsilon-\varepsilon_{ij}} (1-\theta_i)^{e_j-\varepsilon+\varepsilon_{ij}}. \tag{6}$$

Often, we consider sufficiently large partitions and the binomial distributions become well-approximated by Gaussians with means and variances as in (5). Hence, ε is well-approximated as the sum of two Gaussian random variables

$$\begin{aligned} p_{\text{arbit}}(\varepsilon|e_i, e_j, n_i, n_j, n) &\simeq \mathcal{N}(\varepsilon|\hat{\varepsilon}^{\text{sym}}(e_i, e_j, n_i, n_j, n), \hat{\sigma}^{\text{sym}}(e_i, e_j, n_i, n_j, n)), \\ \text{with} \quad \hat{\varepsilon}^{\text{sym}}(e_i, e_j, n_i, n_j, n) &= \tfrac{e_i n_j + e_j n_i}{n-1}, \\ \hat{\sigma}^{\text{sym}}(e_i, e_j, n_i, n_j, n) &= \tfrac{e_i n_j (n-n_j-1) + e_j n_i (n-n_i-1)}{(n-1)^2}. \end{aligned} \tag{7}$$

Assuming a knn graph with, at least on average, $e_i = kn_i$ and $e_j = kn_j$, this simplifies further

$$\begin{aligned} \hat{\varepsilon}^{\text{sym}}_{n_i,n_j,n} &:= \hat{\varepsilon}^{\text{sym}}(kn_i, kn_j, n_i, n_j, n) = \tfrac{2kn_i n_j}{n-1} \\ \hat{\sigma}^{\text{sym}}_{n_i,n_j,n} &:= \hat{\sigma}^{\text{sym}}(kn_i, kn_j, n_i, n_j, n) = \tfrac{kn_i n_j (2n-n_i-n_j-2)}{(n-1)^2}. \end{aligned} \tag{8}$$

1.1.3 Comparing model predictions with sampling based estimates for kNN graphs

To assess how well models (2) and (7) capture the distribution of the number of inter-edges ε in sampling-based simulations, we studied three sampling-based models for bipartitioned graphs. The first model randomly connects half-edges in a bipartitioned set of nodes to simulate constant-outdegree $k = 1$ graphs with partition sizes n_i and $n_j = n - n_i$. The second and third model fit kNN graphs to data sampled from a Gaussian and proceed with randomly partitioning this graph by assigning nodes to random binary partition label — again for fixed n_i and $n_j = n - n_i$. The third model is equivalent to the second but uses instead of the non-symmetric kNN graph, the symmetrized kNN graph — as results, for instance, in UMAP from the fuzzy union of local simplicial sets to each data point [14].

Results of the sampling simulations based on estimates of 1000 samples show the following findings (Supplemental Figure S1).

1. There is a strong difference in the functional form of distributions for constant and arbitrary degree graphs for small numbers of nodes ($n = 10$ $n_i = 4$). For higher values ($n = 100$, $n_i = 40$), both distributions approach Gaussians (two left panels of Supplemental Figure S1).

2. The constant-degree sampling based estimate agrees well with the prediction for the constant-degree model (2), the kNN-fitting based sampling estimate agrees well with the prediction of the arbitrary-degree model (7) (two left panels of Supplemental Figure S1).

3. If one evaluates the kNN graph as in UMAP, one obtains a an empirical distribution that is no longer well-described by (7) (center panels of Supplemental Figure S1).

4. The number of inter-edges depends on the partition-sizes (right panel of Supplemental Figure S1). The relation on n_i and $n_j = n - n_i$ can be seen to be quadratic from (2).

As mentioned, kNN graphs do not have constant-degree k distributions as nearest-neighbor-relations among two observations $\boldsymbol{x}_{\iota_1}$ and $\boldsymbol{x}_{\iota_2}$ are often not symmetric. Still kNN graphs are somewhat "close" to a constant degree k distribution. Supplemental Figure S1 provides strong evidence that this is also the case when using model (7), which, in principle, accounts for arbitrary degree distributions. This can also be theoretically understood by acknowledging that also in the model, strong deviations from degree k are unlikely, in particular for sparse graphs with $k \ll n$ which implies a comparatively low variance of k.

1.1.4 Statistical test for disconnectedness and PAGA connectivity measure

Given equation (7), it is straight-forward to write down a hypothesis test for disconnectedness of two partitions i and j with the null hypothesis that edges of i and j are randomly connected among each other. One can reject the hypothesis of connectedness at a p-value p with an observed inter-edge number $\varepsilon_{ij}^{\text{sym}}$, observed edge numbers of partitions e_i, e_j and partition sizes n_i, n_j

$$p = \int_0^{\varepsilon_{ij}^{\text{sym}}} d\varepsilon \, p_{\text{arbit}}(\varepsilon | e_i, e_j, n_i, n_j, n) \simeq \text{erf}\Big(\frac{\varepsilon_{ij}^{\text{sym}} - \hat{\varepsilon}(e_i, e_j, n_i, n_j, n)}{\hat{\sigma}(e_i, e_j, n_i, n_j, n)} \Big). \tag{9}$$

In order to define a connectivity measure for a PAGA graph, this p-value has the desired property of varying in $[0, 1]$ taking large values if it is likely that partitions are connected and taking small values if it is unlikely. Given that we expect the null model of random connections to strongly overestimate inter-edge numbers when applied in practice, the exponential variation of the p-value hampers interpretation and visualization of PAGA graphs, in which edge thickness should indicate connectivity. Using the p-values logarithmized version resolves the exponential variation, but does not vary in $[0, 1]$ anymore.

So, instead of using the p-value for quantifying connectivity c of partitions in a PAGA graph, we suggest a linear function of the test statistic

$$c_{ij} = a \Big(\frac{\varepsilon_{ij}^{\text{sym}} - \hat{\varepsilon}(e_i, e_j, n_i, n_j, n)}{\hat{\sigma}(e_i, e_j, n_i, n_j, n)} + b \Big) \tag{10}$$

such that the connectivity measure takes values in $[0, 1]$ with $\varepsilon = 0$ corresponding to connectivity $c = 0$ and $\varepsilon \geq \hat{\varepsilon}$ corresponding to connectivity $c = 1$. Solving these conditions for a and b results in

$$c_{ij} = \begin{cases} \frac{\varepsilon_{ij}^{\text{sym}}}{\hat{\varepsilon}^{\text{sym}}(e_i, e_j, n_i, n_j, n)} & \text{if } \varepsilon_{ij}^{\text{sym}} < \hat{\varepsilon}^{\text{sym}}(e_i, e_j, n_i, n_j, n) \\ 1 & \text{else.} \end{cases} \tag{11}$$

This provides the basic model for "PAGA connectivity" through this paper — below we discuss how this measure could presumably be improved.

1.1.5 Relation to modularity

In practice we are not interested in random partitions but in partitions that show stronger intra-partition than inter-partition connectivity. Typically, one uses modularity optimization [11, 15, 16] to compute such a partitioning. To conform with our previous notation, we not only give modularity m_{ij} but also a symmetrized version m_{ij}^{sym} of it

$$m_{ij} = \varepsilon_{ij} - \hat{\varepsilon}^{\text{mod}}(e_i, e_j), \quad m_{ij}^{\text{sym}} = m_{ij} + m_{ji} = \varepsilon_{ij}^{\text{sym}} - 2\hat{\varepsilon}^{\text{mod}}(e_i, e_j), \quad \hat{\varepsilon}^{\text{mod}}(e_i, e_j) = \frac{e_i e_j}{e_i + e_j}, \tag{12}$$

where e_i is the number of outgoing edges of nodes in partition i, ε_{ij} is the number of edges from partition i to partition j and $\varepsilon_{ij} = \varepsilon_{ij} + \varepsilon_{ji}$ is the number of inter-edges, as before. Note that in

modularity optimization algorithms, the modularity measure is only evaluated for the same partition $i = j$. Here, $\hat{\varepsilon}^{\mathrm{mod}}(e_i, e_j)$ is the expected number of inter-edges between partitions when randomly connecting edges to edges — and not edges to nodes as in (7). This results in a probability $\theta_j = \frac{e_j}{e}$ for connecting a given edge in partition i to an edge in partition j, hence $e_i\theta_j$ expected edges from i to j. If one assumes a directed constant-outdegree k kNN graph with $e_i = kn_i$, $\hat{\varepsilon}^{\mathrm{sym}}(e_i, e_j)$ and $2\hat{\varepsilon}^{\mathrm{mod}}(e_i, e_j)$ agree up to a small difference in the denominator — $(e_i + e_j - k)$ versus $(e_i + e_j)$ — which comes from avoiding self-loops in $\hat{\varepsilon}^{\mathrm{sym}}(e_i, e_j)$ — a property of kNN graphs fitted to data.

1.1.6 Feature-space based connectivity

Let us now investigate whether we can relate the graph-based statistical measures of connectivity to a notion of connectivity for the feature space $\mathcal{X}$ of observations $\boldsymbol{x}_\iota$. This will also provide the basis for systematically benchmarking the previous graph-based measures on simulated data.

Consider the Gaussian mixture

$$p(\boldsymbol{x}) = \frac{1}{2}(\mathcal{N}(\boldsymbol{x}) + \mathcal{N}(\boldsymbol{x} - \boldsymbol{\delta})), \tag{13}$$

where $\mathcal{N}(\boldsymbol{x})$ is the standard normal distribution ($\boldsymbol{\mu}_1 = \mathbf{0}$, $\boldsymbol{\Sigma} = \mathrm{diag}(\mathbf{1})$) and $\boldsymbol{\mu}_2 = \boldsymbol{\delta}$ denotes the mean of a second shifted normal distribution. Data sampled from this model show two clusters if the distance between the cluster centers $\delta = |\boldsymbol{\delta}|$ is large enough. Otherwise, upon visual inspection, the data "appear to be connected" even though model selection on Gaussian mixture models might still select (13) as the model that most likely explains the data. While the structure of the model (13) should be considered topologically disconnected as it does not rely on the parametrization of a connected manifold, clearly, a kNN-graph fitted to data sampled is strongly connected across clusters if the cluster centers are close enough.

Can we define a notion of connectivity on the level of the model that reflects the connectivity observed in the knn graph? We suggest to define a "connected region" of the model as a subset of its support in which it is not possible to determine the cluster origin of a given sample with confidence higher than ϵ, that is

$$C = \{\boldsymbol{x} \,|\, p(\boldsymbol{x}|\boldsymbol{\mu} = \boldsymbol{\mu}_1) - p(\boldsymbol{x}|\boldsymbol{\mu} = \boldsymbol{\mu}_2) < \epsilon\}. \tag{14}$$

We can then consider clusters as connected if the "connected region" has probability mass greater than some threshold α:

$$p_C = \int_C d\boldsymbol{x}\, p(\boldsymbol{x}) > \alpha, \tag{15}$$

hence p_C provides a measure of connectivity that measures how likely one observes "unassignable points" or "connecting points" when sampling from the cluster model. The corresponding empirical estimator for $\{\boldsymbol{x}_\iota\}$ and n observations

$$\hat{p}_C = \frac{|\{\boldsymbol{x}_\iota | \boldsymbol{x}_\iota \in C\}|}{n}, \tag{16}$$

provides a measure for the connectivity of the empirical distribution, given the model assumption. Clearly, this is not yet a confidence measure for connectivity as arises from a hypothesis test. Testing the null hypothesis that *clusters are disconnected* requires to fix the parameter δ: then one can test whether the fraction of "connecting points" $\hat{p}_C$ is significantly higher than p_C as predicted the null model — if this is the case, one judges that the data is connected. However, fixing a parameter δ to some value can only be done in an ad-hoc way. Considering the alternative of testing the null hypothesis that *cluster centers are identical* does not provide an answer that uses the wished notion of connectivity. Finally, testing the null hypothesis that *clusters are connected* would require to specify a model class that models connectivity accurately, which would require a manifold-based model with continuous latent variables that parametrize the manifold. To circumvent these problems, we further investigate the estimator $\hat{p}_C$ as a direct measure for connectivity.

In particular, we want to compare $\hat{p}_C$ as arises from a cluster model with the subset of inter-cluster edges in the corresponding kNN graph. In the case of example (13), this amounts to determining the probability mass of the connected region

$$p_C = \int_{\frac{\delta}{2}-x_\epsilon}^{\frac{\delta}{2}+x_\epsilon} dx_{\boldsymbol{\delta}} \int d\boldsymbol{x}_{\perp\boldsymbol{\delta}}\, p(\boldsymbol{x}), \tag{17}$$

where points on the hyperplane $\perp \frac{\boldsymbol{\delta}}{2}$ fulfill $p(\boldsymbol{x}|\boldsymbol{\mu} = \boldsymbol{0}) - p(\boldsymbol{x}|\boldsymbol{\mu} = \boldsymbol{\delta}) = 0$ and x_ϵ determines how "thick" the "connected region" subspace around this hyperplane is. It is determined according to (14),

$$\int_{\frac{\delta}{2}}^{\frac{\delta}{2}+x_\epsilon} dx_{\boldsymbol{\delta}} \int d\boldsymbol{x}_{\perp\boldsymbol{\delta}} \mathcal{N}(\boldsymbol{x}) = \int_{\frac{\delta}{2}}^{\frac{\delta}{2}+x_\epsilon} dx_{\boldsymbol{\delta}} \int d\boldsymbol{x}_{\perp\boldsymbol{\delta}} \mathcal{N}(\boldsymbol{x}-\boldsymbol{\delta}) - \epsilon. \tag{18}$$

This can be easily solved by exploiting the radial symmetry of the integrand for the second term in $\boldsymbol{x} = x_{\boldsymbol{\delta}} + \boldsymbol{x}_{\perp\boldsymbol{\delta}}$ in the $d-1$ dimensional subspace. Using additionally the simple form of the isotropic Gaussian

$$S_d \int_0^\infty d\rho \rho^{d-2} \mathcal{N}(\rho) \int_{\frac{\delta}{2}}^{\frac{\delta}{2}+x_\epsilon} dx_{\boldsymbol{\delta}} \mathcal{N}(x_{\boldsymbol{\delta}}) = S_d \int_0^\infty d\rho \rho^{d-2} \mathcal{N}(\rho) \int_{\frac{\delta}{2}}^{\frac{\delta}{2}+x_\epsilon} dx_{\boldsymbol{\delta}} \mathcal{N}(x_{\boldsymbol{\delta}} - \delta) - \epsilon \tag{19}$$

where $S_d = \frac{(d-1)\pi^{\frac{d-1}{2}}}{\Gamma(1+\frac{d-1}{2})}$. While this cannot be solved in closed form, the expression could be trivially further simplified using the error function erf. However, note the following.

While points that fall in the "connected region" surely qualify as inter-partition edges of the kNN graph that is partitioned according to the cluster labels, the assumption of a constant "thickness" x_ϵ of the corresponding subspace is incompatible with kNN graphs. For large δ and large ρ, i.e. large distance of the "connected region" from the cluster centers, data points are more sparsely sampled and nearest neighbors are further separated. Hence, inter-cluster edges in a kNN graph correspond to different uncertainties ϵ about cluster membership depending on the value of δ and ρ and one has to assume $x_\epsilon \equiv x_\epsilon(\delta, \rho)$. As a rough approximation, we estimate (17) using $x_\epsilon \propto x_\epsilon^{\text{const}}(\delta + \rho)$ and obtain, assuming $x_\epsilon^{\text{const}}$ is small,

$$p_C \simeq \int_{\frac{\delta}{2}-x_\epsilon}^{\frac{\delta}{2}+x_\epsilon} dx_{\boldsymbol{\delta}} \int d\boldsymbol{x}_{\perp\boldsymbol{\delta}} \mathcal{N}(\boldsymbol{x}) \propto \delta^2 \mathcal{N}(\delta). \tag{20}$$

1.1.7 Benchmarks for Louvain-partitioned kNN graphs for clustering data

Let us study the result of connectivity measures (11) and (20) when applied to the Gaussian mixture model (13) in 20 dimensions. We consider 500 randomly generated datasets $\{\boldsymbol{x}_\iota\}$ of 100 observations $\boldsymbol{x}_\iota$. We consider both random partitions and Louvain partitions for increasing cluster distance δ in (13). The result is shown in Supplemental Figure S2:

a, Random partitions lead to the parabolic form of the number inter-edges predicted by (11) and the PAGA connectivity measure c is observed to vary between 0.5 and 1. The observed variance of c is the variance of the rescaled $\varepsilon/\hat{\varepsilon}_{n_i,n_j,n}$ sum of binomial variables, hence decreases as a square root for increasing values of n_i.

b, Louvain partitioning a kNN graph deviates considerably from the random model already for cluster distance $\delta = 0$, which is expected as the number of inter-edges is optimized to a local minimum. However, also in this case, the PAGA connectivity measure c is observed to be approximately independent of partition size n_i, hence, it continues to correct for the variation of ε with respect to partition sizes n_i. This shows that — while the null model of random partitions is unsuitable for Louvain partitions — dealing with Louvain partitions can be accounted for with a mere offset or threshold in the test statistic c. As this threshold is hard to estimate a priori, it gives rise to the only numerical parameter in the implementation of the PAGA plotting function: the 'threshold' for c.

c, The summary statistics shows that PAGA connectivity c is distributed with a much lower relative variance as compared to the number of inter-edges ε. However, this statement only holds for the outer quartiles of the distribution, the box plots show that the inner-quartiles of the distribution of ε and c are comparable.

d, Comparing the graph-based measures with the feature-space based measure of the frequency of points falling into a "connecting region", one observes the expected qualitative agreement.

While Supplemental Figure S2c shows that the connectivity measure c of (11) has the desired property of showing low variance by virtue of the corrected partition-size effect, it would be desirable to have null model that correctly estimates the observed number of inter-edges of a Louvain-partitioned graph at cluster distance $\delta = 0$. Evidently, such a model cannot be obtained as a simple expression but has to be fitted to data or obtained by sampling-based techniques. Independent of the computational burden introduced by this, which could hamper an efficient application in practice, there are many open conceptual problems of how to estimate the null model for real data, which is beyond the scope of this paper.

Let us finally inspect an example with several partitions sampled from a Gaussian mixture with five cluster centers. It can be seen that connectivity of clusters shows meaningful variation and reflects the basic assumption that a fixed number of inter-edges for small cluster gives higher confidence in its connection than for a large cluster (Supplemental Figure S3).

Supplemental Note 1.2: PAGA for mapping transitions between partitions

In this section, we briefly outline the generalization of the PAGA idea of abstracting from single-cell neighborhood relations to relations among groups by discarding insignificant relations attributed to a noisy graph.

In the context of RNA velocity [17], consider again a kNN graph in d-dimensional feature space $\{\boldsymbol{x}_\iota\} = \mathcal{X} = \mathbb{R}^d$, given a distance measure δ such as Euclidean distance. Fitting a model for the steady state of reaction dynamics from unspliced to spliced RNA for each gene allows to define a velocity vector $\boldsymbol{v}_\iota \in \mathbb{R}^d$ for each cell ι. By computing the projection of the velocity vector onto the directions between the k neighbors of the cell in the kNN graph, it is possible to define a weight matrix W with entries

$$w_{\iota_1\iota_2} = \frac{(\boldsymbol{x}_{\iota_1} - \boldsymbol{x}_{\iota_1}) \cdot \boldsymbol{v}_{\iota_1}}{|\boldsymbol{x}_{\iota_1} - \boldsymbol{x}_{\iota_1}|}. \tag{21}$$

The resulting directed graph provides indication for that a cell transitions from node ι_1 to ι_2 with a transition tendency or strength $w_{\iota_1\iota_2}$. We note that W is not a stochastic matrix but simply the weighted adjacency matrix of a directed graph — hence we use the convention of adjacency matrices where $w_{\iota_1\iota_2}$ is associated with an edge pointing from ι_1 to ι_2. For a stochastic matrix, one usually follows the opposite convention (row vectors of probabilities and right stochastic matrices).

In order to judge whether a given group of cells i shows a significant drift or only random transitions to another group of cells j, we first need to correct for the group sizes n_i and n_j. Intuitively, one requires more transitions from i to j if n_i is large. In order to correct for the size effect, we again consider the expected number of inter-edges from i to j under random sampling as in (7). This time, however, we do not consider the symmetrized version $\varepsilon_{ij}^{\text{sym}}$ but consider ε_{ij}. The estimator that is corrected for n_i hence reads

$$v_{ij} = \frac{\sum_{\iota_1 \in i, \iota_2 \in j} w_{\iota_1\iota_2}}{\varepsilon_{ij}}, \text{ where } \varepsilon_{ij} = \frac{e_i n_j}{n-1}. \tag{22}$$

This is in complete analogy to (11) only that here, we consider edge weights that deviate from one.

To judge whether the corrected summed difference of transition tendencies

$$v_{ij}^{\text{diff}} = v_{ij} - v_{ji} \tag{23}$$

provide significant evidence for transitions to one group — deviates from 0 — we use a t-test. If v_{ij}^{diff} takes a positive significant value, we orient an arrow in the PAGA graph from i to j. The negative log p-value of the test has the desired property of taking large values if we can be confident in transitions from i to j. However, it approaches infinity for large values of w_{ij}^{diff}, which is undesirable. Instead of the p-value, therefore, we use the summed transitions v_{ij}^{diff} normalized to the standard deviation of the $n_{v_{ij}} = |\{1|v_{\iota_1\iota_2} \neq 0 \vee v_{\iota_2\iota_1} \neq 0\}|$ observations of

$$v_{\iota_1\iota_2}^{\text{diff},i,j} = \begin{cases} v_{\iota_1\iota_2} & \text{if } v_{\iota_1\iota_2} \neq 0 \\ -v_{\iota_2\iota_1}, & \text{if } v_{\iota_2\iota_1} \neq 0, \end{cases} \quad \forall \iota_1 \in i, \iota_2 \in j. \tag{24}$$

Hence, $v_{ij}^{\text{diff}} = \mu(v_{\iota_1\iota_2}^{\text{diff},i,j})$ and $\sigma_{ij}^{\text{diff}} = \sigma(v_{\iota_1\iota_2}^{\text{diff},i,j})$ and we define the PAGA transition tendency as

$$\tilde{v}_{ij} = \frac{v_{ij}^{\text{diff}}}{\sigma_{ij}^{\text{diff}}}. \tag{25}$$

Examples are shown in Figure 3. We note that a completely different approach to modeling transitions between partitions has been proposed by David and Averbuch [18]. We also note that the approach here does not suffer from the problems discussed in [19]. Regarding the general interpretation of single-cell trajectories on snapshot data, we refer the reader to [5] and [19].

Supplemental Note 1.3: PAGA for multi-resolution analysis of data

Consider the finite node set V of observations of cells. To define a multi-scale graph, we assume an additional filtration $\{V^{(i)}\}_i$ on V, i.e. $V^{(i)}$ being a partition or clustering of V, with at its lowest level $i = 0$ being the whole set $V^{(0)} = \{V\}$, and at its highest $i = n$ being the set of nodes $V^{(n)} = \{\{v_\iota\}|\iota \in V\}$, such that for $i > 0$

$$\forall W \in V^{(i)} \exists W' \in V^{(i-1)} : W \subset W'. \tag{26}$$

We interpret a low filtration level i as a coarse-grained clustering of observations, which means a low-resolution representation of the topology of data. Going to higher resolutions i, we aim to describe more fine-grained aspects of the data and eventually, for $i = n$, the single-cell level. Usually we are only interested in a small set of coarse-grained resolutions $\{i_1, i_2\}$ that have a meaningful biological interpretation.

We can describe the filtration more explicitly by $V^{(i)} = \{W_1^{(i)}, \ldots, W_{m_i}^{(i)}\}$, where by definition $W_1^{(i)} \sqcup \ldots \sqcup W_{m_i}^{(i)} = V$. Then the partial order of the filtration induces a map

$$f^{(i)} : \{1, \ldots, m_i\} \to \{1, \ldots, m_{i-1}\} \tag{27}$$

such that

$$W_j^{(i)} \subset W_{f^{(i)}(j)}^{(i-1)}. \tag{28}$$

We can concatenate this to get for $i' < i$

$$f^{(i,i')} := f^{(i')} \circ f^{(i'+1)} \circ \ldots \circ f^{(i)}, \tag{29}$$

which lets us map any cluster on level i to a lower resolution i'.

Beyond Figure 3 and Figure 4, Supplementary Figure S4 shows a particularly simple example for a multi-resolution embedding for the simulated data of Figure 2. Supplemental Figure S4a and b show partitioned single-cell graphs and the associated PAGA graphs at two resolutions that differ from the one in Figure 2. Supplemental Figure S4c and d visualize the map (29) between clusters at different resolutions via association matrices. Supplemental Figure S4e shows the single-cell graph colored with mapped partitions.

Within PAGA, we combine this with an additional connectivity structure between vertices (v_1, v_2). We therefore assume a given graph $G = (V, E)$ on V with edges $e = \{v_1, v_2\}$ such that $v_1 \neq v_2 \in V$. The edges may possibly be weighted with a function $w(e)$ or be directed (v_1, v_2).

The filtration $V^{(i)}$ induces coarse-grained graphs $G^{(i)} = (V^{(i)}, E^{(i)})$, where multiple definitions of coarse-grained edge sets could make sense. For instance, one could define the "complete abstracted graph" for resolution i by identifying $E^{(n)} := E$ and demanding

$$e = \{w_j, w_k\} \in E^{(i)} \leftrightarrow \{W^{(i-1)}_{f^{(i)}(j)}, W^{(i-1)}_{f^{(i)}(k)}\} \in E^{(i-1)}, \tag{30}$$

That is, any two supersets are connected if there is a connection one level below and hence on any level below. This is theoretically attractive since it is transitive, but is problematic in practice. It is not robust for noisy graphs and leads to strongly connected coarse-grained graphs that reflect the exact connectivity of the single-cell graph.

PAGA solves this by generating weighted coarse-grained graphs, which are then "abstracted" by thresholding low-weight edges. The simplest weight would be the corresponding number of inter-edges in the single-cell graph. Within PAGA, we use a statistical model to derive the weight (11) as the number of inter-edges in the single-cell graph divided by the expected number of inter-edges when assuming random connections (Supplemental Note 1.1).

Given an abstracted graph $\{G^{(i)}\}$ as defined above, we can now study paths across resolutions. We say that a low-resolution path $p^{(i-1)} = (w^{(i-1)}_{j_1}, \dots, w^{(i)}_{j_k})$ on $G^{(i-1)}$ represents a high-resolution path p^i on G^i if

$$\{w^{(i)}_{k_l}, w^{(i)}_{k_{l+1}}\} \in E^{(i)}, \quad k_l = f^{(i)}(j_l), \quad k_{l+1} = f^{(i)}(j_l + 1). \tag{31}$$

Conversely, we say that a high-resolution path $p^{(i)} = (w^{(i)}_{j_1}, \dots, w^{(i)}_{j_k})$ on $G^{(i)}$ is represented by a low-resolution path $p^{(i-1)}$ on $G^{(i-1)}$ if

$$\{w^{(i-1)}_{f^{(i)}(j_l)}, w^{(i-1)}_{f^{(i)}(j_l+1)}\} \in E^{(i-1)}. \tag{32}$$

Note that the complete abstracted graph represents all high-resolution paths. However, a "good abstraction" of a high-resolution graph represents many high-resolution paths while being as sparse as possible. Sparsity arises naturally by demanding that the abstracted graph only represents those high-resolution paths that are statistically well supported, which is achieved through (11). This also defines the sense in which an abstracted graph can be said to be "topology preserving".

Supplemental Note 1.4: Robustness of PAGA

Let us take a more practical view on the question of whether the topology of two abstracted graphs G_1^* and G_2^* agree under the constraint that the node labels of G_1^* and G_2^* are consistent with each other. Moreover, instead of only detecting exact matches, we aim for a continuous measure of agreement.

Associating a partitioning with a reference partitioning.

To establish such a measure, we first compute the overlaps of the partitions labelled by G_1^* and by G_2^* (Supplemental Figure S4a, b). By that, we generate non-unique associations between partitions, as visualized in an association matrix (Supplemental Figure S4c). The association matrix can either be normalized with respect to the reference groups V_1^* (Supplemental Figure S4c), with respect to the new groups V_2^* (Supplemental Figure S4d) or with respect to the union of partitions, which leads to the Jaccard index. Instead of the Jaccard index we want a score that measures how well two partitions mutually overlap — are mutually contained in each another — and consider the minimum of both mentioned normalizations — the "minimal overlap" — for each combination of groups $(i_1, i_2) \in (V_1^*, V_2^*)$. Supplemental Figure S4e colors each partition in V_1^* with the partition in V_2^* with which it has the largest minimal overlap.

Comparing paths in abstracted graphs.

For each shortest path between two leaf nodes in G_2^*, there is a shortest path between the associated nodes in G_1^*. This enables to compare the two paths and to count the fraction of steps that are consistent among two paths. To measure the agreement of the topologies between two abstracted graphs, we compute the fraction of agreeing steps and the fraction of agreeing paths over all combinations of leaf nodes in two given abstracted graphs.

For instance, consider the shortest path between leafs (21, 2) in the reference graph G_1^* and the shortest path between leafs (7, 11) in the new graph G_2^* in Supplemental Figure S4a and b, respectively:

$$\begin{aligned} p_1 &= (21, 8, 18, 7, 9, 2), \quad p_1 \in G_1^* \\ p_2 &= (7, 2, 9, 10, 11), \quad p_2 \in G_2^*. \end{aligned} \tag{33}$$

By computing the overlap of reference partitions with new partitions, we can map p_1 to the label space of G_2^*

$$p_1^{\text{mapped}} = ((7, 2), (6, 7, 2), (2, 7), (2, 9), (9, 10, 3), (11, 10)), \tag{34}$$

that is, partition 21 in G_1 has finite minimal overlap with partitions 7 and 2 in G_2, partition 8 in G_1 has overlap with partitions 6, 7 and 2 in G_2, and so on.

Transitioning through path p_2 and counting for each transition whether it's present or not in p_1^{mapped} allows to count the number of agreeing steps. If all steps agree with each other, the paths p_1 and p_2 agree with each other. In the example of equation (33), p_2 involves 4 steps, 4 of which agree with p_1^{mapped}.

Benchmark.

In Supplemental Figure S5, we use the just-described measure to demonstrate robustness of PAGA.

A related measure from the literature.

Previously, it has been suggested to correlate the distribution of path lengths of all paths through trees as a measure for topological similarity of trees [4]. Specifically, for a tree whose nodes label sets of data points, the lengths of all paths between all pairs of data points are computed. The correlation of such path-length sets obtained for two trees is suggested as a measure for topological similarity of the two trees. Besides being highly redundant and costly to compute, the resulting measure is very rough as it does not map paths onto each other; that is, it does not account for inconsistencies of paths with the same length.

Supplemental Note 1.5: Remarks on generating and partitioning single-cell graphs

At the heart of PAGA lies the assumption that the single-cell graph G — the kNN graph of observations $\boldsymbol{x}_\iota$ in some feature space — provides a meaningful representation of data. This assumption is on one hand based on the community's success with graph-based clustering [11, 12, 20], pseudotime inference [5], visualization [21, 22] and tSNE [23, 24]. On the other hand, it is based on the observation that neighborhood graphs robustly generalize any local distance measure to a global scale. As any fixed distance measure can at best encode a very rough notion of biological similarity with an exploding error for large distances, it is more robust to only evaluate it locally, and construct the global distances from the graph of neighborhood relations. See how some of us discuss this in more detail in Supplemntal Note 3 of Reference [25].

In this paper, we only consider established preprocessing steps [22, 26, 27] for single-cell transcriptomic data, each of which give rise to a different fixed distance measure. For single-cell imaging data. we consider a learned distance measure as induced by the feature space of a deep learning model [28]. Any other distance measure, for example, the kernel-based measure of Reference [29], or autoencoder representations [30, 31] would also be a viable option. Finally, we remark that denoising the kNN graph is another step, which should be considered. This can for instance be done by "pruning" [12] of by computing neighborhood relations in the truncated spectral approximation of the graph's adjacency matrix ("diffusion map" representation).

A partitioning of G that maximizes the ratio of intra- to inter-partition edges is natural in the sense that it reveals regions of the graph with different connectivity and hence, different topology.

Optimizing this ratio is known as optimizing modularity [15]. An efficient algorithm for this [11] has been suggested for single-cell biology by Levine *et al.* [12]. Loosely speaking, one expects to obtain the clearest coarse-grained group structure of data at a fixed resolution if choosing the partitioning that maximizes modularity. The original implementation of the Louvain algorithm could lead to disconnected communities when nodes were assigned to a common community with a single node connecting two or more parts of this community. When the central node was reconsidered by the algorithm and moved to a different community, a disconnected community remained. This unexpected behaviour could be fixed by splitting disconnected communities before each community aggregation step in the implementation of [32]. Note that the Louvain algorithm has also been adopted by popular single-cell analysis toolkits such as Seurat [26] and Cell Ranger [27]. Many other possibilities for partitioning G — or clustering the data — exist: we mention spectral clustering and the graph-based hierarchical clustering [33], which is based on a random-walk based distance measure.

Supplemental Note 1.6: Remarks on the reconciliation of clustering with trajectory inference algorithms

Here, we provide a more formal explanation of the discussion of Figure 1 in the main text. The aim of any pseudotime of given data is to provide a continuous latent variable that associates with continuous variation in the data; presumably the process that generated the data. Furthermore, pseudotemporal ordering of cells enables the identification of the relative timing of different events during the process — it tries to represent the internal "clock" of cells as encoded in its molecular configuration. A clustering analysis, by contrast, relates neither cells nor clusters to each other. With the PAGA graph G^*, which describes the connectivity and "continuity" relations c_{ij} of clusters i and j of G, and the pseudotime measure $d(\iota_1, \iota_2)$, which measures the continuous progression of a cell ι_1 to a cell ι_2, one reconciles the result of a clustering analysis with the aim of a pseudotime analysis: Each cluster is related to any other cluster as either being disconnected or connected with one or several paths of high confidence in G^*. Moreover, within each cluster, each cell is ordered according to pseudotime. One can hence trace a continuous process from a root cell ι_{root} in a root cluster i_{root} to any terminal cell ι_{end} in its terminal cluster i_{end} by following a path of high confidence $(i_{\text{root}}, i_1, i_2, \ldots, i_{\text{end}})$ in G^*. In each step of this path, the pseudotemporal ordering provides an ordering with single-cell resolution and, hence, one traces the progression of single cells along an ensemble of paths of high-confidence in G. Thereby, PAGA provides a topology preserving map of cells as (G^*, d). Without the PAGA graph G^*, computing the ensemble of highly confident paths from from i_{root} to ι_{end} in G is a computationally much harder and an unsolved problem — only recently, during the revision of this paper, a simulation-based approximative approach has been proposed, but not validated on many datasets [34]. Presumably, the heuristics for their inference are less transparent and easy to control than the heuristics involved in partitioning a graph G and generating a PAGA G^*.

Supplemental Note 2: Random walks on graphs

On the single-cell level, the continuity of connections are believed to be well parametrized by a "pseudotime" [35, 36] that measures the distance covered in a continuous progression along a manifold. A robust kernel-based measure that can be easily extended to a graph, diffusion pseudotime, has recently been proposed by Haghverdi *et al.* [5]. This measure and similar scale-free random-walk based measures though do not account for clustering structure in the data; they are undefined for disconnected graphs. Below, we show how to overcome this limitation by extending these measures.

Interpreting random walks and their path distributions.

It is important to note that in the whole paper, when we say "random walk on a graph", we mean a discrete-space Markov process on the state space given by the nodes of the graph and non-zero transition probabilities between any two connected nodes.

Such random walks can be used to probe the global topology of the single-cell graph G but do not provide a good model for the biological processes that one might hypothesize to have generated the data in the first place. The primary deficiencies of the Markov random walk when seen as a model for a biological process are the following.

- Undirectedness. When progressing along a differentiation trajectory, at some point, one expects commitment of a cell to a specific fate and a directed motion to that fate with some fluctuations. By contrast, the diffusive motion induced by the Markov random walk is highly non-directed, which leads to unrealistic paths that go back and forth and pass through remote regions of the graph.
- Independence of the expression of specific genes. The random walk is independent of the expression of specific genes, which may be quite relevant for the commitment to specific fates; it only depends on global differences in the transcriptome.

These deficiencies of the random walk become apparent already when modeling a biological process using the simple stochastic differential equation based model discussed in Supplemental Note 5.3.

The distribution of single-cell paths that correspond to a path through the abstracted graph, by contrast, resolves the problem of undirectedness by bounding the distribution to the ribbon of the connected sequence of groups.

Existing random-walk based distance measures.

For a single-cell graph G with n_{nodes} nodes and n_{edges} edges, consider the normalized graph laplacian [37, 38]

$$L = I - T, \quad T = D^{-1}A, \tag{35}$$

where I is the $n_{\text{nodes}} \times n_{\text{nodes}}$ identity matrix and T is the transition matrix of the same shape. T is obtained from the weighted adjacency matrix A of G by normalizing with row sums of A, that is, D is the diagonal matrix that stores the degree of each node in G. In practice, we compute the weights of the adjacency using a Gaussian decay with euclidian distance between two data points in gene expression space, see e.g. Reference [5]; after that, we density-normalize obtained weights [39, 40] as in Reference [5].

For a study of random walks generated by T, a spectral analysis of L and T is convenient and one hence considers the matrices $\widetilde{L}$ and $\widetilde{T}$, which are obtained by multiplying (35) with $D^{-\frac{1}{2}}$ from the left and with $D^{\frac{1}{2}}$ from the right

$$\widetilde{L} = I - \widetilde{T}, \quad \widetilde{T} = D^{\frac{1}{2}} T D^{-\frac{1}{2}}. \tag{36}$$

Hence, L and $\widetilde{L}$ have the same spectrum $\{1-\lambda_1, 1-\lambda_2, \dots\}$ and the spectrum of T and $\widetilde{T}$ is given as $\{\lambda_1, \lambda_2, \dots\}$ with $\lambda_1 = 1, \lambda_2 < \lambda_1, \dots$ for a connected graph G [37, 38]. For a disconnected graph with n_{comps} disconnected components, the adjacency matrix A has block-diagonal form with n_{comps} blocks and there are n_{comps} eigenvalues λ_r with value 1 and corresponding eigenvectors that are the indicator vectors of the connected components. The eigenvectors $\widetilde{v}$ of $\widetilde{T}$ are related to the right eigenvectors v of T as [37–39]

$$v_{r\iota} = \frac{\widetilde{v}_{r\iota}}{\sqrt{D_{\iota\iota}}} \qquad \forall r, \iota. \tag{37}$$

The right eigenvectors v of T are known as "diffusion map" coordinates [39], whereas the left eigenvectors span the space of probability distributions of configurations of the Markov process. The first right eigenvector, corresponding to $\lambda = 1$, is the all-one vector — with only 1 as entry — and the first left eigenvector is the stationary state of the Markov process.

Using this notation, one obtains the mean commute time — the average number of steps one needs to arrive from node ι_1 to another node ι_2 — in equation (38a) [37, Corollary 3.2]. One obtains

"diffusion distance" [33, 39] in equation (38b) and "diffusion pseudotime" [5] in equation (38c).

$$\text{mean commute time}(\iota_1, \iota_2) = 2n_{\text{edges}} \sum_{r=2}^{n_{\text{nodes}}} \left(\frac{1}{1-\lambda_r}\right)^2 (v_{r\iota_1} - v_{r\iota_2})^2, \tag{38a}$$

$$\text{diffusion distance}^2_{(k)}(\iota_1, \iota_2) = \sum_{r=2}^{n_{\text{nodes}}} \lambda_r^{2k} (v_{r\iota_1} - v_{r\iota_2})^2, \tag{38b}$$

$$\widetilde{\text{dpt}}^2(\iota_1, \iota_2) = \sum_{r=2}^{n_{\text{nodes}}} \left(\frac{\lambda_r}{1-\lambda_r}\right)^2 (\tilde{v}_{r\iota_1} - \tilde{v}_{r\iota_2})^2, \tag{38c}$$

$$\text{dpt}^2(\iota_1, \iota_2) = \sum_{r=2}^{n_{\text{nodes}}} \left(\frac{\lambda_r}{1-\lambda_r}\right)^2 (v_{r\iota_1} - v_{r\iota_2})^2, \tag{38d}$$

$$\text{algebraic distance}_{(k)}(\iota_1, \iota_2) = \sum_{r=1}^{r_{\text{max}}} (\chi^{(k)}_{r\iota_1} - \chi^{(k)}_{r\iota_2})^2, \quad \chi^{(k)}_r = L^k \chi^{(0)}_r. \tag{38e}$$

With equation (38d), we give a slightly altered definition of diffusion pseudotime, which is consistent with the other measures. Highly related is algebraic distance on the graph as given in (38e), see e.g., [40].

Interpretation of random-walk based distance measures.

Random-walk based distances on graphs have first been used to cluster graphs in Reference [33] (38b) and Reference [41] (38a), albeit without considering neighborhood graphs of data points. Reference [39] proposed "diffusion distance" for measuring the similarity between data points, albeit not on a graph, but for a Gaussian kernel matrix. Then, a random-walk based distance measure for single-cell data has first been proposed to measure the similarity between cells by Reference [5]; again not formulated for graphs. These authors introduced the measure of equation (38c), which integrates out the number of steps n_{steps} in (38b) to arrive at a scale-free measure.

The dpt measure is highly similar to (38a), which is easier to interpret and scale-free, too: it measures the average number of steps it takes to walk from ι_1 to ι_2. While equation (38b) arises as the summed difference of transition probabilities to all other nodes for two random-walks of length n_{steps} that start at nodes ι_1 and ι_2, respectively [33, 39], (38d) considers the sum over all numbers of n_{steps}, hence a difference of "accumulated transition probabilities", which are difficult to interpret; the interpretation of equation (38c) is not easier.

Algebraic distance, which has been used for graph partitioning in recent years [40], approximates (38a) and diffusion pseudotime and provides the computationally most efficient way of computing a random-walk based distance measure.

Random-walk based distance measures for disconnected graphs.

Evidently, both scale-free distance measures, mean commute time (38a) and diffusion pseudotime (38c), are not defined for a disconnected graph G for which $n_{\text{comps}} > 1$ eigenvalues are 1: they yield an infinite distance even for two nodes ι_1 and ι_2 that are in the same connected component of G. It is important to realize that each connected component of G automatically leads to a block T_b in the transition matrix T that is itself a valid transition matrix and the spectrum of T is the union of the spectra of the blocks T_b. The eigenvectors of T are the eigenvectors of the blocks T_b filled with zeros at the positions of the other blocks [see e.g. 38]. Hence, we propose to extend mean commute time and diffusion pseudotime for disconnected graphs as

$$\text{mean commute time}(\iota_1, \iota_2) = 2n_{\text{edges}} \sum_{r=n_{\text{comps}}+1}^{n_{\text{nodes}}} \left(\frac{1}{1-\lambda_r}\right)^2 (v_{r\iota_1} - v_{r\iota_2})^2, \tag{39a}$$

$$\widetilde{\text{dpt}}(\iota_1, \iota_2) = \sum_{r=n_{\text{comps}}+1}^{n_{\text{nodes}}} \left(\frac{\lambda_r}{1-\lambda_r}\right)^2 (\tilde{v}_{r\iota_1} - \tilde{v}_{r\iota_2})^2 + \sum_{r=1}^{n_{\text{comps}}} (\tilde{v}_{r\iota_1} - \tilde{v}_{r\iota_2})^2. \tag{39b}$$

$$\text{dpt}(\iota_1, \iota_2) = \sum_{r=n_{\text{comps}}+1}^{n_{\text{nodes}}} \left(\frac{\lambda_r}{1-\lambda_r}\right)^2 (v_{r\iota_1} - v_{r\iota_2})^2, \tag{39c}$$

The distribution of zeros in the eigenvectors v_r and $\tilde{v}_r$ guarantees that for two nodes ι_1 and ι_2 in the same connected component b, only the spectrum of the block transition matrix T_b contributes. For two nodes ι_1 and ι_2 in two disconnected components, the measures take the sum of their maximum values in both components, which should be interpreted as infinite. Without problem, one can make this explicit in the equations by distinguishing cases in which ι_1 and ι_2 belong to the same component from cases in which they belong to different components.

We note that, in practice, instead of summing over all eigenvectors n_{nodes}, we sum over a low number of eigenvectors — "diffusion components" in the language of Coifman *et al.* [39] — as others [5, 41].

While in the present publication, we use equation (39b) throughout, we expect that equation (39a) could be useful in the future due to its easier interpretation.

Supplemental Note 3: Comparisons with previous approaches

Establishing fair comparisons with other algorithms is difficult mainly for two reasons. (i) Comparisons on real data are problematic as a quantitative undebatable ground truth is hard to obtain. (ii) It is very easy to "make algorithms fail", for example, by choosing an unsuitable preprocessing or pathologic parameters. Hence, after a comparison of concepts (Supplemental Note 3.1), we restrict ourselves to addressing fundamental problems and qualitatively wrong predictions of other algorithms for simple, simulated minimal examples with known ground truth (Supplemental Note 3.2), well-known real data, where we were able to reproduce published testing conditions [2] (Supplemental Note 3.3) and a comparison of runtimes (Supplemental Note 3.4). We note that a recent comprehensive trajectory inference review [42] positively mentions PAGA.

Supplemental Note 3.1: Conceptual comparisons

Graph abstraction

PAGA provides a graph abstraction method that is suitable for deriving interpretable abstractions of the noisy kNN-like graphs that are typically used to represent the manifolds arising in scRNA-seq data. The review of Hu & Shi [43] discusses graph abstraction within the context of (1) graph coarsening, (2) loss-less topology compression and (3) community detection.

Within graph coarsening, graphs are coarsened using "micro-clustering" approaches, such as edge-collapsing [44], which do not yield interpretable abstractions. Graph coarsening is typically used as a preprocessing step for graph partitioning (community detection) or for initializing force-directed graph drawing. This procedure parallels one use case of PAGA: initializing an embedding algorithm based on an embedding of a PAGA graph, see Methods.

Lossless topology compression algorithms perform exact compression through motifs. Hu & Shi state that even though "lossless compression of large graphs can be critical to understand the details of the original graph, it is extremely difficult to achieve on real graphs with small world nature". Using such approaches, we were unable to derive meaningful results for scRNA-seq data.

Community detection algorithms themselves are not yet "graph abstraction" algorithms, as they do not provide a method for defining edges among the detected communities. Closest to PAGA is the reference [45], who use a modularity-based hierarchical graph. However, the "edge bundling" methods therein are "pattern-based" and not statistical and hence, unsuitable for noisy scRNA-seq data.

Algorithms used in biology

Monocle 2 [2] uses "reversed graph embedding" [46], which aims to fit a geometrical model for a graph to projections of the data to a low-dimensional latent space. Even though, in principle, any model could be used for that, in practice, only tree-like models are computationally tractable. Hence, Monocle 2 tries to force data into a tree-like topology without providing a statistical measure for how reliable the resulting fit is.

Spade [47], StemID 2 [3], Eclair [4], TSCAN [48] and Mpath [49] use different clustering algorithms such as k-means, k-medoids, hierarchical clustering or DBSCAN in a dimensionality-reduced space.

In a second step, they fit a minimum spanning tree to either the centroid or medoid distances or to projections of cells on linear connections between centroids or medoids. In this, distances are computed using simple, fixed distance measures such as the euclidean or the correlation-based distance. Neither do these distances between clusters measure how well and if clusters are connected with each other, nor do these methods try to invoke a statistical model to address this question. The computationally expensive sampling procedures in StemID 2 and Eclair only partially alleviate the principle problem of high non-robustness that is caused by these deficiencies. Projections on linear connections between clusters assume a linear geometry of differentiation trajectories, which is certainly violated in practice. Hence, Mpath, for example, has only been shown to reconstruct processes with a single branching [49]. Moreover, it is important to note that none of the used clustering algorithms in these methods guarantees a topology preserving coarse-graining of the data: disconnected regions of data might cluster together and connected regions might be torn apart.

DPT [5] computes a random-walk based pseudotime for all cells. It cannot handle data with disconnected structure and is only able to detect single branchings which, in addition, is prone to violating the topological structure in the data (see, e.g., Figure 2c of Reference [13]). This problem becomes particularly pronounced in the extension of DPT to multiple branchings [6].

Rizvi *et al.* [13] suggest topological data analysis (TDA), in particular, the MAPPER algorithm [10] for analyzing single-cell data. MAPPER constructs a partial coordinatization of the data in the form of a simplicial complex, which has some similarity with the PAGA graph introduced in the present work. Both MAPPER's simplicial complex and the PAGA graph represent connectivity of clusters in the data. However, the construction of MAPPER's simplicial complex differs fundamentally from the PAGA graph. In particular, the clusters do not correspond to regions with controlled resolution and high intra-connectivity in the kNN graph, which are typically used in the field as proxies for cell types or cell states. Hence, in contrast to PAGA, MAPPER does not use an easily interpretable partitioning of the data into connected and disconnected regions, but a highly fine-grained, overlapping clustering, where clusters merely serve a technical purpose and are computed on a very low-dimensional map of data. Moreoever, MAPPER's connectivity measure directly reflects the amount of overlap between these clusters. Hence, the measure does not induce a natural simplification by discarding statistically insignificant connections - it retains the full connectivity information of the overlapping clustering. Very generally, MAPPER is *not* based on simplifying a kNN graph of data points but uses the mentioned low-dimensional representation of data. Hence, MAPPER does not allow for a robust, random-walk-on-the-kNN-graph based distance measure for pseudotime estimation. Even though TDA allows the definition of continuous coordinates on the simplicial complex, their robustness and interpretability has not been shown. We interpret PAGA as a pragmatic, easily-interpretable, scalable and robust way of performing topological data analysis.

The graph coarsening approach of Wagner *et al.* [50] — developed at the same time and independently of PAGA — is also based on computing a connectivity measure based on the number of inter-edges between clusters in the single-cell graph. However, the approach has only been validated on a single dataset and does not provide a ready-to-use computational method for users. In addition, their metric computes for each pair of partitions the ratio of the number of inter-edges versus the number of the union of their out-going edges, which systematically overestimates connectivity. Assume two partitions share, relative to their size, a very small number of edges with each other and none with any other cluster - likely, these edges are a result of noise and the clusters are actually disconnected. However, in the approach of Wagner *et al.*, such clusters appear as strongly connected.

The hierarchical tSNE approach of Unen *et al.* [51] — published during the revision of the present paper — presents an idea for measuring "overlap between influence regions" of clusters obtained from density-based clustering. However, the measure is not related to the measure developed for PAGA. The authors proceed in using this overlap as a similarity measure to implement a hierarchical version of tSNE.

The graph-based approach p-Creode of Herring *et al.* [52] — published during the revision of the present paper — uses a density-adjusted kNN graph to produce an ensemble of potential trajectories. A consensus graph is then selected from the ensemble using a graph similarity metric.

We do not compare our method to Wishbone [53], which can only detect a single branching, nor to the fundamentally different, fully supervised approach STEMNET [54].

Supplemental Note 3.2: Simulated minimal examples with known ground truth

We consider a minimal example with known ground truth to show that graph abstraction overcomes qualitative conceptual problems in the design of algorithms for the inference of lineage trees. The dataset consists in a connected tree-like manifold and two disconnected clusters and has a clearly defined ground truth — a computational model for hematopoiesis (Supplemental Figure S11, Supplemental Note 5.3) — and very little noise. Nonetheless, none of Monocle 2, StemID 2, Eclair and DPT produce sensible results. Only when we removed the clusters from the data, these algorithms made sensible predictions. To reproduce the following comparisons and to get more information follow this link.

Graph abstraction recovers the ground truth (Supplemental Figure S6a). Monocle 2 [2] — even after testing several values for the latent-space dimension in Monocle 2 [2] — fits a tree to the clusters and misses to recognize the continuous manifold in the data (Supplemental Figure S6b). D. Grün ran stemID 2 — the unpublished successor of stemID [3] — on the minimal example. However, the produced graph-like object erroneously connects one of the clusters with the manifold (Supplemental Figure S6c). For the minimal example, we could not produce any sensible result neither with Eclair [4] — even after optimizing parameters in correspondence with the author G. Giecold — nor DPT [5].

As a control, we aimed to obtain sensible results with the competing algorithms and considered a simpler dataset that only contains the continuous tree-like manifold of the previous example. Graph abstraction recovers the ground truth (Supplemental Figure S7a). Monocle 2 can be tuned — by adjusting the latent space dimension — to yield the correct result (Supplemental Figure S7b). Eclair [4] obtains a wrong result even for this simple tree (Supplemental Figure S7c, d). DPT [5] does, by construction, not infer a lineage tree but merely detects two branching subgroups; similar to a clustering algorithm. In a hierarchical implementation [6], it detects an arbitrary number of groups. Using the latter to detect four branchings we can detect two branchings (Supplemental Figure S7e) but fail to detect a third. Note that only when using diffusion maps for visualization, the clustering of groups appears natural (Supplemental Figure S7f).

Supplemental Note 3.3: Hematopoiesis

Comparisons for data of Paul et al. [1].

In the recent Monocle 2 paper of Qiu *et al.* [2] the data of Paul *et al.* [1] served as an example for the reconstruction of a complicated differentiation tree in Supplemental Figure 16. In the preprocessing step for the analysis of this data, Qiu *et al.* removed a cluster of lymphoid cells. In many situations, clusters of cells might not be annotated or not be clearly disconnected and it might not be clear whether one should remove them from the data. We therefore wondered what would happen when rerunning Monocle 2 with the exact same settings on the same data but keeping the cluster of lymphoids. While PAGA produces the same result irrespective of the presence of this cluster — it is simply disconnected in Figure 2, Monocle 2's inferred tree changes dramatically and displays qualitatively wrong biology, for instance, by placing the lymphoid cluster in the center of the myeloid differentiation.

Comparison for data of Nestorowa et al. [7].

Supplemental Figure S9 shows a comparison for data of Reference [7].

Supplemental Note 3.4: Runtimes

The authors of Monocle 2 report a runtime of 9 min for 8 000 cells [55] and a linear scaling. Extrapolation yields 76.5 min for 68 000 cells for which PAGA takes a few seconds — constructing the neighborhood graph and running clustering take an additional 3 min; hence PAGA is about 25 times faster.

PAGA for 1.3 million cells runs 90 s — constructing the neighborhood graph and running clustering takes about 45 min each. No other trajectory inference algorithm scales to such high cell numbers.

Supplemental Note 4: Faithfulness of embeddings to global topology

Consider the cost function of the widely used tSNE algorithm [23],

$$\begin{aligned} p_{\iota\iota'} &= \frac{p_{\iota'|\iota} + p_{\iota|\iota'}}{2N}, \quad p_{\iota'|\iota} = \frac{\exp(-d(\boldsymbol{x}_\iota, \boldsymbol{x}_{\iota'})^2/2\sigma_\iota^2)}{\sum_{\kappa\neq\iota}\exp(-d(\boldsymbol{x}_\iota, \boldsymbol{x}_\kappa)^2/2\sigma_\iota^2)}, \quad p_{\iota\iota} = 0, \\ q_{\iota\iota'} &= \frac{(1 + \|\boldsymbol{y}_\iota - \boldsymbol{y}_{\iota'}\|^2)^{-1}}{\sum_{\kappa\neq\lambda}(1+\|\boldsymbol{y}_\kappa - \boldsymbol{y}_\lambda)\|^2)^{-1}}, \quad q_{\iota\iota} = 0, \\ \mathrm{KL}(P||Q) &= \sum_{\iota\iota'} p_{\iota\iota'} \log\frac{p_{\iota\iota'}}{q_{\iota\iota'}}. \end{aligned} \tag{40}$$

The double sum over ι and ι' is implemented as a sum over edges $e = (\iota, \iota')$ in the kNN graph of high-dimensional observations $\boldsymbol{x}_\iota \in \mathcal{X}$. In the language of this paper, we say that $p_e \equiv p_{\iota\iota'}$ quantifies the connectivity of node ι with ι' in the high-dimensional space $\mathcal{X}$ and $q_e \equiv q_{\iota\iota'}$ quantifies the connectivity in the embedding space $\mathcal{Y}$. The optimized cost function hence is

$$\mathrm{KL}(P||Q) = \sum_{e\in E_{\mathcal{X}}} p_e \log\frac{p_e}{q_e}, \tag{41}$$

where we use the notation $E_{\mathcal{X}}$ to indicate that the edge set entering the optimization has been obtained as a kNN graph in $\mathcal{X}$.

Embedding cost functions as a binary classification problem.

Let us take a different view on the quantification of how faithful the low-dimensional representation $\{\boldsymbol{y}_i\}$ in $\mathcal{Y}$ is to the topology of the high-dimensional representation $\{\boldsymbol{x}_i\}$ in $\mathcal{X}$. Let us define the ground-truth of this classification problem to be the kNN graph $G_{\mathcal{X}} = (V, E_{\mathcal{X}})$ fitted in $\mathcal{X}$. The state space of the classification problem is given by the edge set E_{fc} of the fully-connected graph $G_{\text{fc}} = (V, E_{\text{fc}})$. We note that this is similar to the procedure introduced by [56].

In this classification setting, we require an embedding algorithm to predict for each edge e in E_{fc} whether it is an element of $E_{\mathcal{X}}$. If it is an element, we assign the label $l_e = 1$ to it, otherwise $l_e = 0$. For each edge, the embedding algorithm makes prediction $l_e = 1$ with probability q_e and $l_e = 0$ with $1 - q_e$. The standard cost function used to train such a classifier is the cross-entropy $H(P,Q)$ or logloss, which is equivalent to the negative log-likelihood of the labels under the model

$$\begin{aligned} H(P,Q) &= -\sum_{e\in E_{\text{fc}}} \sum_{l_e\in\{0,1\}} p_e \log(q_e), \\ &= \sum_{e\in E_{\text{fc}}} p_e \log\left(\frac{1}{q_e}\right) + (1-p_e)\log\left(\frac{1}{1-q_e}\right). \end{aligned} \tag{42}$$

By virtue of $\mathrm{KL}(P,Q) = H(P,Q) - H(P)$ and $H(P) = -\sum_{e\in E_{\text{fc}}} p_e$, the KL divergence of predicted distribution Q and reference distribution P is

$$\begin{aligned} \mathrm{KL}(P||Q) &= \sum_{e\in E_{\text{fc}}} \sum_{l_e\in\{0,1\}} p_e \log\left(\frac{p_e}{q_e}\right) \\ &= \sum_{e\in E_{\text{fc}}} p_e \log\left(\frac{p_e}{q_e}\right) + (1-p_e)\log\left(\frac{1-p_e}{1-q_e}\right). \end{aligned} \tag{43}$$

As in the optimization of the cost-function, the reference distribution P is fixed, it does not matter whether cross entropy or KL divergence is optimized.

Let us continue with interpreting the KL divergence, which both appears in UMAP and tSNE — however, in the case of tSNE the second term in (43) is absent. We can interpret the two terms in the KL divergence as follows

$$\underbrace{p_e \log\left(\frac{p_e}{q_e}\right)}_{\substack{\text{cost of "false negatives",}\\ \text{i.e., disconnected regions}}} \quad \text{and} \quad \underbrace{(1-p_e)\log\left(\frac{1-p_e}{1-q_e}\right)}_{\substack{\text{cost of "false positives"}\\ \text{i.e., overlapping regions}}}. \tag{44}$$

The first term generates cost that can be attributed to false negatives: edges in $E_\mathcal{X}$ that are not "detected" by the embedding algorithm and hence miss in the predicted edge set $E_\mathcal{Y}$. This occurs when a pair of points (ι, ι') is far apart in the embedding space and hence has low or zero predicted connectivity $q_{\iota\iota'}$ but has high connectivity $p_{\iota\iota'}$ in the reference space $\mathcal{X}$. As the cost function diverges if any $q_e = 0$ if the corresponding $p_e \neq 0$, such disconnected structure should in theory not occur. However, it is well-known that tSNE produces many spurious disconnected structures in the embedding — this can be attributed to the fact that values for q_e have to be clipped to finite values so that the cost function itself remains finite and can be numerically stably optimized. Also UMAP [14] suffers from this problem.

The second term in (43) can be attributed to false positives: edges predicted by the embedding algorithm even though they miss in $E_\mathcal{X}$. This occurs in "overlapping regions" of the embedding, where q_e is close to 1 even though p_e is close to 0. This phenomenon is frequently encountered in graph drawing algorithms such as ForceAtlas2 [57].

A novel cost function that accounts for global topology.

Both disconnected and overlapping structure in the embedding present strong violations of the global topology represented by $G_\mathcal{X}$ that hinder interpretability by humans. However, in the cost function (43), these violations only contribute as strongly as violations of local topology with a weight of order

$$\frac{1}{|E_\mathcal{X} \cup E_\mathcal{Y}|} \lesssim \frac{1}{kn}, \tag{45}$$

where the right-hand-side estimate holds for kNN graphs. Hence, for high numbers of observations n, the cost of violating global topology approaches zero, even though this is in stark contrast to what is desirable to the human interpreter.

In order to remedy this discrepancy, we suggest a weighted KL (or cross-entropy), which reflects the desire that edges that violate the global topology carry a higher weight than edges that violate local topology. Specifically, we suggest

$$\begin{aligned} \mathrm{KL}_{\text{geo}}(P||Q) &= \mathrm{KL}_{\text{geo}}^{\text{disc}}(P||Q) + \mathrm{KL}_{\text{geo}}^{\text{overl}}(P||Q) \\ &= \sum_{e \in E_{\text{fc}}} \underbrace{\frac{d_e^q}{d_e^p} p_e \log\left(\frac{p_e}{q_e}\right)}_{\text{disconnected cost}} + \underbrace{\frac{d_e^p}{d_e^q}(1-p_e)\log\left(\frac{1-p_e}{1-q_e}\right)}_{\text{overlapping cost}}, \end{aligned} \tag{46}$$

where d_e^p and d_e^q denote random-walk based distances in the kNN graphs $G_\mathcal{X}$ and $G_\mathcal{Y}$ and are hence estimators of geodesic distances of the manifolds in $\mathcal{X}$ and $\mathcal{Y}$. See an extensive review of such distances in Supplemental Section 2.

Clearly, geodesic distance captures important aspects of the global topology of a manifold. The interpretation of the factors $\frac{d_e^q}{d_e^p}$ and $\frac{d_e^p}{d_e^q}$ is hence as follows. If there is a globally disconnected region in the embedding, this causes d_e^q to diverge to infinity. If the region is also disconnected in the high-dimensional reference space, the effect cancels out in $\frac{d_e^q}{d_e^p}$, otherwise, the violation receives a high weight in (43). The argument is analogous for overlapping regions.

PAGA provides faster convergence and more interpretable single-cell embeddings.

Throughout this paper, established manifold learning algorithms only provided embeddings that would violate the topology of data found in the high-dimensional feature space, see for instance

Figure 3. Using (46), we can now quantify these violations and show that PAGA-initialized manifold learning both provides embeddings that are more faithful to the global topology and allows faster convergence also with respect to the conventional cost function (43). The results are summarized in Supplemental Figure S10) for the first two examples shown in Figure 2.

1. The two rows showing different embeddings highlight globally disconnected points (marked as D0, D1, ...) and globally overlapping points (marked as O0, O1, ...) identified by maximizing the weight factors $\frac{d_e^q}{d_e^p}$ and $\frac{d_e^p}{d_e^q}$, respectively. The title of the embeddings show the conventional KL divergence and the reweighted geodesic $\mathrm{KL}_{\mathrm{geo}}$ introduced in (46). Quantitative values agree with the visual impression except for the FA embedding of Paul *et al.*, which we would expect to have a higher $\mathrm{KL}_{\mathrm{geo}}^{\mathrm{overl}}$.

2. The two rows showing statistics of KL measures for different embeddings and with or without initialization with PAGA have been produced by rerunning embedding algorithms 10 times. They show that either PAGA+FA or PAGA+UMAP achieve the best values throughout. The right-most panel shows KL values after early stopping, illustrate that already after 50 optimization epochs, KL values comparable to the converged result ($\geq$200 epochs) are obtained.

Supplemental Note 5: Datasets

Supplemental Note 5.1: Simulated dataset for hematopoiesis

We use a literature-curated qualitative – boolean – gene regulatory network of 11 genes that aims to describe myeloid differentiation [58] and has been used for benchmarking the reconstruction of gene regulatory network from a single-cell graph of state transitions in Reference [59]. The boolean network evolves according to

$$
\begin{aligned}
Gata2 &= Gata2 \ \wedge \ \neg(Gata1 \ \wedge \ Fog1) \ \wedge \ \neg Pu.1, \\
Gata1 &= (Gata1 \ \vee \ Gata2 \ \vee \ Fli1) \ \wedge \ \neg Pu.1, \\
Fog1 &= Gata1, \\
EKLF &= Gata1 \ \wedge \ \neg Fli1, \\
Fli1 &= Gata1 \ \wedge \ \neg EKLF, \\
SCL &= Gata1 \ \wedge \ \neg Pu.1, \\
Cebpa &= Cebpa \ \wedge \ \neg(Gata1 \ \wedge \ Fog1 \ \wedge \ SCL), \\
Pu.1 &= (Cebpa \ \vee \ Pu.1) \ \wedge \ \neg(Gata1 \ \vee \ Gata2), \\
cJun &= Pu.1 \ \wedge \ \neg Gfi1, \\
EgrNab &= (Pu.1 \ \wedge \ cJun) \ \wedge \ \neg Gfi1, \\
Gfi1 &= Cebpa \ \wedge \ \neg EgrNab.
\end{aligned}
\tag{47}
$$

These boolean equations are translated into ordinary differential equations following Reference [8]. Within Scanpy [6], they are simulated as stochastic differential equations by adding Gaussian noise.

Simulations result in four classes of realizations of gene expression time series, each of which corresponds to the convergence to an attractor that represents a certain cell fate of myeloid progenitors: erythrocyte, neutrophil, monocyte and megakaryocyte (Supplemtal Figure S11). We concatenate four typical realizations (Supplemtal Figure S11c, d) with 160 time steps, which yields 640 data points in total.

To model clustering, we sample 640 data points from a Gaussian mixture model with two Gaussians and random centers in an 11-dimensional space. The minimal dataset of Figure 2 and Supplemental Figure S11 consists of the concatenated data matrices of the simulated myeloid progenitor development data and the Gaussian mixture model, corresponding to 1280 cells.

Supplemental Note 5.2: One million neurons

As an input for the PAGA analysis, we used the kNN graph obtained by running the *pp.recipe_zheng17* [27] preprocessing function within Scanpy [6] and computing neighbors on 50 principal components. See Supplemental Figure S12 for visualizations of these data using PAGA and UMAP.

Supplemental Note 5.3: Experimental datasets for hematopoiesis

For preprocessing the data of Paul *et al.* [1], we used Scanpy's preprocessing function *pp.recipe_zheng17*, for the data of Nestorowa *et al.* [7], we used *pp.recipe_weinreb17*. For the data of Dahlin *et al.* [60], we used the preprocessing of the original publication. We then computed kNN graphs on 20 principal components with $k = 4$ for Paul *et al.* and Nestorowa *et al.* and for $k = 7$ for Dahlin *et al.*, as in the original publication. PAGA can be applied on the resulting kNN graphs and yields meaningful results. However, for Figure 2, we further denoised the graph by approximating its adjacency matrix with the first 15 spectral components. We performed this approximation by recomputing a kNN graph using the first 15 diffusion components of the PCA-based graph. For this recomputation of the kNN graph, we used $k = 10$ for Paul *et al.* and Nestorowa *et al.* and for $k = 15$ for Dahlin *et al.*. We note that denoising the kNN graph by a different technique has already been suggested in Reference [12].

See Supplemental Figure S13 for an example of annotating clusters using PAGA for Paul *et al.* [1].

Supplemental Note 5.4: Planaria

For the analysis of the Planaria data of [25], we used the preprocessing of these authors. We computed a kNN graph on 30 principle components with 30 neighbors.

Supplemental Note 5.5: Zebrafish embryo

As an input for the PAGA analysis, we used the kNN graph and clustering of Wagner *et al.* [50] as provided by the authors.

Supplemental Note 5.6: Deep-learning-processed image data

Without extensive preprocessing, the graph of neighborhood relations of data points in gene expression space is useless if computed with a simple fixed distance metric (euclidian, cosine, correlation-based, etc.). If one considers the pixel space of images the problem is even worse and it is impossible to come up with preprocessing methods that lead to a meaningful distance metric. It has recently been shown that a deep learning model can generate a feature space in which distances reflect the continuous progression of cell cycle and a disease [28], that is, deep learning can generate a feature space in which data points are positioned according to biological similarity and by that generates a distance metric that is much more valuable than a simple fixed distance metric. We demonstrate that graph abstraction is useful for reconstructing the cell cycle from image data while and identifying a cluster of damaged cells (Supplementary Figure S14).

References

[1] Paul, F. *et al.* Transcriptional heterogeneity and lineage commitment in myeloid progenitors. *Cell* **163**, 1663–1677 (2015).

[2] Qiu, X. *et al.* Single-cell mRNA quantification and differential analysis with census. *Nature Methods* **14**, 309 – 315 (2017).

[3] Grün, D. *et al.* De novo prediction of stem cell identity using single-cell transcriptome data. *Cell Stem Cell* **19**, 266–277 (2016).

[4] Giecold, G., Marco, E., Garcia, S. P., Trippa, L. & Yuan, G.-C. Robust lineage reconstruction from high-dimensional single-cell data. *Nucleic acids research* **44**, e122–e122 (2016).

[5] Haghverdi, L., Büttner, M., Wolf, F. A., Buettner, F. & Theis, F. J. Diffusion pseudotime robustly reconstructs branching cellular lineages. *Nature Methods* **13**, 845–848 (2016).

[6] Wolf, F. A., Angerer, P. & Theis, F. J. SCANPY: large-scale single-cell gene expression data analysis. *Genome Biology* **19** (2018).

[7] Nestorowa, S. *et al.* A single-cell resolution map of mouse hematopoietic stem and progenitor cell differentiation. *Blood* **128**, e20–e31 (2016).

[8] Wittmann, D. M. *et al.* Transforming boolean models to continuous models: methodology and application to t-cell receptor signaling. *BMC Syst. Biol.* **3**, 98 (2009).

[9] 10X Genomics. 1.3 million brain cells from E18 mice.

[10] Singh, G., Mémoli, F. & Carlsson, G. E. Topological methods for the analysis of high dimensional data sets and 3d object recognition. In *Eurographics Symposium on Point-Based Graphics* (2007).

[11] Blondel, V. D., Guillaume, J.-L., Lambiotte, R. & Lefebvre, E. Fast unfolding of communities in large networks. *J. Stat. Mech.* **2008**, P10008 (2008).

[12] Levine, J. H. *et al.* Data-driven phenotypic dissection of AML reveals progenitor–like cells that correlate with prognosis. *Cell* **162**, 184–197 (2015).

[13] Rizvi, A. H. *et al.* Single-cell topological rna-seq analysis reveals insights into cellular differentiation and development. *Nature Biotechnology* **35**, 551–560 (2017).

[14] McInnes, L. & Healy, J. Umap: Uniform manifold approximation and projection for dimension reduction. *arXiv* 1802.03426 (2018).

[15] Newman, M. E. J. & Girvan, M. Finding and evaluating community structure in networks. *Phys. Rev. E* **69**, 026113 (2004).

[16] Newman, M. E. J. Modularity and community structure in networks. *Proceedings of the National Academy of Sciences* **103**, 8577–8582 (2006).

[17] La Manno, G. *et al.* Rna velocity of single cells. *Nature* **560**, 494 (2018).

[18] David, G. & Averbuch, A. Hierarchical data organization, clustering and denoising via localized diffusion folders. *Applied and Computational Harmonic Analysis* **33**, 1–23 (2012).

[19] Weinreb, C., Wolock, S., Tusi, B. K., Socolovsky, M. & Klein, A. M. Fundamental limits on dynamic inference from single-cell snapshots. *Proceedings of the National Academy of Sciences* **115**, E2467–E2476 (2018).

[20] Xu, C. & Su, Z. Identification of cell types from single-cell transcriptomes using a novel clustering method. *Bioinformatics* **31**, 1974–1980 (2015).

[21] Fruchterman, T. M. J. & Reingold, E. M. Graph drawing by force-directed placement. *Software: Practice and Experience* **21**, 1129–1164 (1991).

[22] Weinreb, C., Wolock, S. & Klein, A. M. Spring: a kinetic interface for visualizing high dimensional single-cell expression data. *Bioinformatics* btx792 (2017).

[23] van der Maaten, L. & Hinton, G. Visualizing data using t-sne. *Journal of Machine Learning Research* **9**, 2579–2605 (2008).

[24] Amir, E.-a. D. *et al.* visne enables visualization of high dimensional single-cell data and reveals phenotypic heterogeneity of leukemia. *Nature Biotechnology* **31**, 545–552 (2013).

[25] Plass, M. *et al.* Cell type atlas and lineage tree of a whole complex animal by single-cell transcriptomics. *Science* **360**, eaaq1723 (2018).

[26] Satija, R., Farrell, J. A., Gennert, D., Schier, A. F. & Regev, A. Spatial reconstruction of single-cell gene expression data. *Nature Biotechnology* **33**, 495–502 (2015).

[27] Zheng, G. X. Y. *et al.* Massively parallel digital transcriptional profiling of single cells. *Nature Communications* **8**, 14049 (2017).

[28] Eulenberg, P. *et al.* Reconstructing cell cycle and disease progression using deep learning. *Nature communications* **8**, 463 (2017).

[29] Wang, B., Zhu, J., Pierson, E., Ramazzotti, D. & Batzoglou, S. Visualization and analysis of single-cell rna-seq data by kernel-based similarity learning. *Nature methods* **14**, 414–416 (2017).

[30] Lopez, R., Regier, J., Cole, M. B., Jordan, M. I. & Yosef, N. Deep generative modeling for single-cell transcriptomics. *Nature methods* **15**, 1053 (2018).

[31] Eraslan, G., Simon, L. M., Mircea, M., Mueller, N. S. & Theis, F. J. Single cell RNA-seq denoising using a deep count autoencoder. *bioRxiv* 300681 (2018).

[32] Traag, V. Louvain. *GitHub* (2017).

[33] Pons, P. & Latapy, M. Computing communities in large networks using random walks. *Computer and Information Sciences - ISCIS* 284 (2005).

[34] Farrell, J. A. *et al.* Single-cell reconstruction of developmental trajectories during zebrafish embryogenesis. *Science* eaar3131 (2018).

[35] Trapnell, C. *et al.* The dynamics and regulators of cell fate decisions are revealed by pseudotemporal ordering of single cells. *Nature Biotechnology* **32**, 381–386 (2014).

[36] Bendall, S. C. *et al.* Single-cell trajectory detection uncovers progression and regulatory coordination in human B cell development. *Cell* **157**, 714–725 (2014).

[37] Lovász, L. Random walks on graphs: A survey. *Combinatorics, Paul Erdös is Eighty* **2**, 1 (1993).

[38] von Luxburg, U. A tutorial on spectral clustering. *Statistics and Computing* **17**, 395 (2007).

[39] Coifman, R. R. *et al.* Geometric diffusions as a tool for harmonic analysis and structure definition of data: Diffusion maps. *Proceedings of the National Academy of Sciences* **102**, 7426–7431 (2005).

[40] Safro, I., Sanders, P. & Schulz, C. Advanced coarsening schemes for graph partitioning (2012).

[41] Fouss, F., Pirotte, A., Renders, J.-M. & Saerens, M. Random-walk computation of similarities between nodes of a graph with application to collaborative recommendation. *IEEE Transactions on knowledge and data engineering* **19**, 355–369 (2007).

[42] Saelens, W., Cannoodt, R., Todorov, H. & Saeys, Y. A comparison of single-cell trajectory inference methods: towards more accurate and robust tools (2018).

[43] Hu, Y. & Shi, L. Visualizing large graphs. *Wiley Interdisciplinary Reviews: Computational Statistics* **7**, 115–136 (2015).

[44] Bartel, G., Gutwenger, C., Klein, K. & Mutzel, P. An experimental evaluation of multilevel layout methods. In Brandes, U. & Cornelsen, S. (eds.) *Graph Drawing*, 80–91 (Springer Berlin Heidelberg, Berlin, Heidelberg, 2011).

[45] Shi, L. *et al.* Himap: Adaptive visualization of large-scale online social networks. In *2009 IEEE Pacific Visualization Symposium*, 41–48 (2009).

[46] Mao, Q., Wang, L., Tsang, I. & Sun, Y. Principal graph and structure learning based on reversed graph embedding. *IEEE Transactions on Pattern Analysis and Machine Intelligence* **PP**, 1–1 (2017).

[47] Qiu, P. *et al.* Extracting a cellular hierarchy from high-dimensional cytometry data with spade. *Nature Biotechnology* **29**, 886–891 (2011).

[48] Ji, Z. & Ji, H. Tscan: Pseudo-time reconstruction and evaluation in single-cell rna-seq analysis. *Nucleic acids research* **44**, e117 (2016).

[49] Chen, J., Schlitzer, A., Chakarov, S., Ginhoux, F. & Poidinger, M. Mpath maps multi-branching single-cell trajectories revealing progenitor cell progression during development. *Nature Communications* **7**, 11988 (2016).

[50] Wagner, D. E. *et al.* Single-cell mapping of gene expression landscapes and lineage in the zebrafish embryo. *Science* eaar4362 (2018).

[51] van Unen, V. *et al.* Visual analysis of mass cytometry data by hierarchical stochastic neighbour embedding reveals rare cell types. *Nature Communications* **8** (2017).

[52] Herring, C. A. *et al.* Unsupervised trajectory analysis of single-cell RNA-seq and imaging data reveals alternative tuft cell origins in the gut. *Cell Systems* **6**, 37–51.e9 (2018).

[53] Setty, M. *et al.* Wishbone identifies bifurcating developmental trajectories from single-cell data. *Nature Biotechnology* **34**, 637–645 (2016).

[54] Velten, L. *et al.* Human haematopoietic stem cell lineage commitment is a continuous process. *Nature Cell Biology* **19**, 271–281 (2017).

[55] Qiu, X. *et al.* Reversed graph embedding resolves complex single-cell trajectories. *Nature methods* **14**, 979–982 (2017).

[56] Venna, J., Peltonen, J., Nybo, K., Aidos, H. & Kaski, S. Information retrieval perspective to nonlinear dimensionality reduction for data visualization. *Journal of Machine Learning Research* **11**, 451–490 (2010).

[57] Jacomy, M., Venturini, T., Heymann, S. & Bastian, M. ForceAtlas2, a continuous graph layout algorithm for handy network visualization designed for the gephi software. *PLoS ONE* **9**, e98679 (2014).

[58] Krumsiek, J., Marr, C., Schroeder, T. & Theis, F. J. Hierarchical differentiation of myeloid progenitors is encoded in the transcription factor network. *PLoS ONE* **6**, e22649 (2011).

[59] Moignard, V. *et al.* Decoding the regulatory network of early blood development from single-cell gene expression measurements. *Nature Biotechnology* **33**, 269–276 (2015).

[60] Dahlin, J. S. *et al.* A single cell hematopoietic landscape resolves eight lineage trajectories and defects in kit mutant mice. *Blood* blood–2017–12–821413 (2018).
